# A branching process model for dormancy and seed banks in randomly fluctuating environments

**DOI:** 10.1007/s00285-021-01639-6

**Published:** 2021-07-19

**Authors:** Jochen Blath, Felix Hermann, Martin Slowik

**Affiliations:** 1 Present Address: Institute of Mathematics, Technische Universität Berlin, Strasse des 17. Juni 136, 10623 Berlin, Germany; 2 Present Address: Mathematical Institute, University of Mannheim, B6, 26, 68159 Mannheim, Germany

**Keywords:** Dormancy, Persistence, Bienaymé–Galton–Watson process, Branching process, Seed bank, Fitness, 60J80, 60K37, 92D25

## Abstract

The goal of this article is to contribute towards the conceptual and quantitative understanding of the evolutionary benefits for (microbial) populations to maintain a seed bank consisting of dormant individuals when facing fluctuating environmental conditions. To this end, we discuss a class of ‘2-type’ branching processes describing populations of individuals that may switch between ‘active’ and ‘dormant’ states in a random environment oscillating between a ‘healthy’ and a ‘harsh’ state. We incorporate different switching strategies and suggest a method of ‘fair comparison’ to incorporate potentially varying reproductive costs. We then use this concept to compare the fitness of the different strategies in terms of maximal Lyapunov exponents. This gives rise to a ‘fitness map’ depicting the environmental regimes where certain switching strategies are uniquely supercritical.

## Introduction

### Biological motivation

Dormancy is an evolutionary trait that comes in many guises and has evolved independently multiple times across the tree of life. In particular, it is ubiquitous among microbial communities. As a general definition, we say that an individual exhibits a *dormancy trait* if it is able to enter a reversible state of vanishing metabolic activity. It has been observed that a large fraction of microbes on earth is currently in a dormant state, thus creating *seed banks* consisting of inactive individuals (see e.g. Lennon and Jones 2011; Shoemaker and Lennon 2018; Lennon et al. 2020 for recent overviews). A common ecological and evolutionary explanation for the emergence of the corresponding complex dormancy traits is that the maintenance of a seed bank serves as a bet-hedging strategy to ensure survival in fluctuating and potentially unfavourable environmental conditions. Recent theory has also shown that dormancy traits can already be beneficial in competing species models in the presence of sufficiently strong competitive pressure for limited resources (even in otherwise stable environments) (see Blath and Tobiás 2020). However, maintaining a dormancy trait is costly and comes with a substantial trade-off: For example, microbes need to invest resources into resting structures and the machinery required for switching into and out of a dormant state, which are then unavailable for reproduction.

Dormancy also has implications for the pathogenic character of microbial communities and plays an important role in human health. For example, dormancy in the form of persister cells can lead to chronic infections, since these cells can withstand antibiotic treatment (Balaban et al. 2004; Fisher et al. 2017; Lewis 2010). Further, dormancy, both on the level of individual cells as well as the tumor level, plays a crucial role in cancer dynamics (Endo and Inoue 2019). In all of the above situations external treatment can be seen as a form of environmental stress for the pathogens.

Hence, improving the conceptual and quantitative understanding of the mechanisms leading to fitness advantages for individuals with a dormancy trait in fluctuating environments, incorporating the potentially different costs of forming active and dormant offspring (and potential reproductive trade-offs due to the maintenance of dormancy traits), seems to be a worthwhile task.

### Deterministic versus stochastic models and known results

In the last two decades, dormancy-related population dynamic modeling based on *deterministic dynamical systems* has expanded rather rapidly, often with a focus on phenotypic plasticity in microbial communities (see e.g. Bär et al. 2002; Balaban et al. 2004; Kussell et al. 2005; Kussell and Leibler 2005; Malik and Smith 2008; Fowler and Winstanley 2018). The important paper by Balaban et al. (2004) for example describes ‘persistence’ (which can be seen as a form of dormancy) as a phenotypic switch, and several of the above papers deal with models incorporating various switching strategies and fluctuating environments. Kussell et al. (2005) consider periodic antibiotic treatment (and also treat a stochastic version of their model via simulation), and Kussell and Leibler (2005) incorporate randomly changing environments, however under the condition that the random changes are slow. Fitness is typically measured in terms of the (maximal) Lyapunov exponents of the underlying dynamical systems, which is often difficult to evaluate analytically in the presence of random environments. Kussel and Leibler approximate the Lyapunov exponent under a ‘slow environment condition’, reducing the model to an essentially one-dimensional system, which is a strategy that we will meet again in different forms later on. These models have been taken up in a mathematical article by Malik and Smith (2008), who provide a set of rigorous results regarding the maximal Lyapunov exponents of dynamical systems explicitly incorporating dormancy, considering both stochastic and responsive switches in (periodically) changing environments. They also compare these to the corresponding results for populations without dormancy trait (so-called ‘sleepless population’). However, for truly random environments, they do not provide exact representations for the maximal Lyapunov exponents and instead give relatively simple (yet useful) bounds.

Recently, also *stochastic (individual-based) models* for seed banks and dormancy have gained increasing attention, in particular in *population genetics* (Kaj et al. 2001; Blath et al. 2015, 2016, 2020). However, these models are mainly concerned with genealogical implications of seed banks and typically require constant population size (without random environment). In stochastic *population dynamics*, while there are interesting recent simulation studies such as Locey et al. (2017), rigorous mathematical modeling and results are still relatively rare. Here, a suitable framework for individual-based seed bank models with fluctuating population size is given by the theory of multi-type branching processes (in random environments). Indeed, dormancy has been described in a brief example in the book by Haccou et al. (2007, Example 5.3) as a 2-type branching process, which served as a motivation for this paper. For *quiescence* (which is a similar concept as dormancy), a multi-type branching-process model has been proposed in Alarcón and Jensen (2011), including a simulation study. On the theoretical side, in the context of *phenotypic switches*, Dombry et al. (2011) and Jost and Wang (2014), again building on motivation from Kussell and Leibler (2005), have developed a branching-process based framework for phenotypic plasticity and obtained interesting rigorous results on the optimality of switching strategies in random environments. However, their set-ups and results, though closely related, do not focus on dormancy, and only partially cover the reproductive and switching strategies that we are going to discuss in Sect. 2.2 (we will explicitly comment on the differences with respect to our model and results as appropriate). They are able to determine Lyapunov exponents in random environments, but similarly to Kussell and Leibler (2005) under a condition which essentially restricts the problem to a one-dimensional system.

### Modeling approach and organization of the present paper

Our approach, motivated by the example in Haccou et al. (2007), is based on a 2-type branching process $(Z_n)=(Z^1_n, Z^2_n)$ with $Z^1_n$ denoting the active and $Z^2_n$ denoting the dormant individuals at time/generation *n*, which we embed in a random environment that is described by a stochastic process $(E_n)$ and governs the respective sequence of random reproductive laws $(Q(E_n))$. As in Kussell and Leibler (2005), Malik and Smith (2008), Dombry et al. (2011), we will discuss results related to both stochastic/spontaneous as well as responsive switching strategies. Further, we also consider mixed strategies. We aim for explicit results under ‘fair comparison’ regarding resource limitations including potentially different costs for active and dormant offspring, and also in comparison with a 1-type branching process without dormancy trait (‘sleepless case’), expressing qualitative and quantitative *fitness advantages* in terms of the maximal Lyapunov exponent.

By providing a model tailored to dormancy in a random environment, we close a gap related to the multi-type branching process models and results provided in Dombry et al. (2011) and Jost and Wang (2014) related to phenotypic switching, which only partially cover our dormancy models and results. We also extend and refine results of Malik and Smith (2008) which explicitly model dormancy in the deterministic dynamical systems case, but with a smaller set of switching strategies and few results for truly random environments. Additionally, we pay particular attention to reproductive costs related to dormancy.

All models and results will be introduced and discussed in Sect. 2. We observe that there are natural parameter regimes under which either the spontaneous or the responsive switching strategies, or even a mixture of strategies, will be fit, while all the others are detrimental. We discuss the corresponding parameter regimes in detail and illustrate them in certain important cases, see e.g. Figs. 3, 4 and 5. This shows that already in our relatively simple random environment (involving only two states), dormancy leads to a rather rich picture regarding the long-term behaviour of the embedded branching processes.

However, while our results will capture several prototypical scenarios corresponding to both stochastic/spontaneous and responsive switching (and mixtures), we are still far from being able to provide a mathematically complete classification in the full space of switching strategies. One theoretical reason for this is that computing the maximal Lyapunov exponent of a random multiplicative sequence of positive matrices, which is the mathematical core of the problem, is infeasible in general (see e.g. Bougerol and Lacroix (1985); Ledrappier (1984) for an overview of the mathematical theory), and works only if the underlying matrices exhibit additional algebraic properties.

Hence, a further aim of this paper is to provide a small review of current methods to compute/estimate maximal Lyapunov exponents. The reason which allows Dombry et al. (2011) and Jost and Wang (2014) to treat the responsive switching regime in their papers is that their assumptions reduce the system to an essentially one-dimensional case, which interestingly has a similar effect as the ‘slow variation assumption’ of Kussell and Leibler (2005), and we will investigate similar cases. While the exponents are easily accessible for the corresponding ‘rank-1 matrices’ (and, of course, scalars), spontaneous/stochastic switching strategies can a priori involve both ‘rank-1’ and ‘rank-2 matrices’. We will see that while special cases of the stochastic switching regime can again be treated with the rank-1 approach, for the general stochastic switching regime involving rank-2 matrices, techniques used in Malik and Smith (2008) are available, which lead at least to bounds on the Lyapunov exponents. We also provide further bounds and estimators. These theoretical considerations can be found in Sect. 3.

Finally, in Sect. 4, we discuss some open questions and potential further steps in modeling and analysis of dormancy and seed banks in random environments, from a somewhat theoretical perspective.

## Models and main results

Recall that for a classical (1-type) Bienaymé–Galton–Watson process, say $X=(X_n)$, it is assumed that individuals die and reproduce independently of each other according to some given common offspring distribution $Q_X$ on ${\mathbb {N}}_0$. We extend this framework by introducing a second component that acts as a reservoir of *dormant* individuals, also referred to as *seed bank*. Moreover, we allow the offspring distribution in each generation *n* to depend on the state of a random environment process $E=(E_n)$. This gives rise to a particular class of 2-type Bienaymé–Galton–Watson processes in random environment that we introduce formally in Definition 2.1 and which will be the main object of study in this paper. However, later on, we will also discuss more general *p*-type branching processes (for $p \ge 1$) in random environments, so that we will first introduce the corresponding general notation, which is standard in the theory of multi-type branching processes.

### Notation

Let $E=(E_n)_{n \in {\mathbb {N}}_0}$ be a stationary and ergodic Markov chain on some probability space $(\Omega , {\mathcal {F}}, {{\,\mathrm{{\mathbb {P}}}\,}})$ taking values in some measurable space $(\Omega ', {\mathcal {F}}')$ and denote by $\pi _E$ its stationary distribution. Such a process will be called *random environment process*. For $p \in {\mathbb {N}}$, we write ${\mathcal {M}}_1({\mathbb {N}}_0^p)$ to denote the space of probability measures on ${\mathbb {N}}_0^p$, and set $\Theta \mathrel {\mathop :}=\{(Q^{1}, \ldots , Q^{p}) : Q^{i} \in {\mathcal {M}}_1({\mathbb {N}}_0^p)\}$. Elements of $\Theta$ will be interpreted as the collection of the *p* *offspring distributions* on ${\mathbb {N}}_0^p$ (one for each type). An infinite sequence $\Pi = (Q(E_1), Q(E_2), \ldots )$ generated by $(E_n)$ and a random variable $Q\!: \Omega ' \rightarrow \Theta$ will be called sequence of *random offspring distributions* with respect to the environment process $(E_n)$. Finally, a sequence of ${\mathbb {N}}_0^p$-valued random variables $Z_0, Z_1, \ldots$ will be called a *p*-*type Bienaymé–Galton–Watson process in random environment* (*p*-type BGWPRE), if $Z_0$ is independent of $\Pi$ and if for each given realization $(e_1, e_2, \dots )$ of *E* (and thus also of $\Pi$) the process $Z = (Z_n)_{n \in {\mathbb {N}}_0}$ is a Markov chain whose law satisfies

$$\begin{aligned} {\mathcal {L}}\big ( Z_{n} \mid Z_{n-1} = z,\, \Pi = (Q(e_1), Q(e_2), \ldots ) \big ) \;=\; {\mathcal {L}}\bigg ( \sum _{i=1}^p \sum _{j=1}^{z^i}\zeta _{j}^{i} \bigg ), \end{aligned}$$

for every $n \in {\mathbb {N}}$ and $z=(z^1, \dots , z^p) \in {\mathbb {N}}_0^p$, where the $(\zeta _j^i : i \in \{1, \ldots , p\}, j \in {\mathbb {N}})$ are independent random variables taking values in ${\mathbb {N}}_0^p$, and for each $i \in \{1, \ldots , p\}$, the $\zeta _1^i, \zeta _2^i, \ldots$ are identically distributed according to $Q^i(e_n)$. In the language of branching processes, if the state of the environment at time *n* is $e_n \in \Omega '$, then each of the $Z_n^i$ individuals of type *i* alive at time *n* produces offspring according to the probability distribution $Q^i(e_n)$, independent of the offspring production of all the other individuals. For notational clarity, we will often write $Q_Z$ to denote the random variable *Q* that is used in the definition of a branching process *Z*.

We are now ready to define the class of branching processes modeling *dormancy*:

#### Definition 2.1

With the above notation (for $p=2$), a 2-type BGWPRE $Z=(Z_n)$ will be called a *Bienaymé–Galton–Watson process with dormancy in random environment* $(E_n)$, abbreviated BGWPDRE, if, ${{\,\mathrm{{\mathbb {P}}}\,}}$-almost surely,

2.1 $$\begin{aligned} Q_Z^2(E_n)[\{(0,0),(1,0),(0,1)\}] \;=\; 1 \qquad \forall \, n \in {\mathbb {N}}_0. \end{aligned}$$

Particles of type 1 are called *active* and particles of type 2 are called *dormant*.

Note that Condition (2.1) ensures that a (dormant) type 2 particle can either switch its state to type 1 (active), remain in the dormant state 2, or die—no other transitions are possible. There is no restriction on the offspring reproduction of active type 1 particles other than the following first moment condition.

Throughout we assume, for ${{\,\mathrm{{\mathbb {P}}}\,}}$-a.e. realization $(e_1, e_2, \ldots )$ of $(E_n)$ and any $n \in {\mathbb {N}}$, that the distribution $Q(e_n) \in \Theta$ is such that the corresponding random variables $\zeta ^i=(\zeta ^1, \ldots , \zeta ^p)$ distributed according to $Q(e_n)$ satisfy ${{\,\mathrm{{\mathbb {E}}}\,}}[\zeta ^i] < \infty$ for all $i \in \{1, \ldots , p\}$. Moreover, we write

$$\begin{aligned} m_n^{i,j} \;\equiv \; m^{i,j}(e_n) \;\mathrel {\mathop :}=\; {{\,\mathrm{{\mathbb {E}}}\,}}\!\big [Z_{n+1}^j \mid Z_n = (\delta _{ik})_k,\, \Pi _n = Q_Z(e_n) \big ]. \end{aligned}$$

to denote the expected number of offspring of type *j* produced by a single particle of type *i* in generation *n* in the environment $Q_Z(e_n)$, and we denote by

2.2 $$\begin{aligned} M_n \;\equiv \; M(e_n) \;\mathrel {\mathop :}=\; (m_n^{i,j})_{i,j} \end{aligned}$$

the corresponding mean matrix. Suppose that, for any $n \in {\mathbb {N}}$, the matrix $M_n$ is irreducible. Then, by the Perron–Frobenius-Theorem, the spectral radius $\varrho _n \equiv \varrho (M_n)$ of $M_n$ is a simple eigenvalue with $|\lambda | \le \varrho _n$ for any (other) eigenvalue $\lambda$ of $M_n$.

### Branching processes with dormancy in constant environment

As a gentle warm-up, we first compare the survival probabilities and extinction times of a classical 1-type BGWP with those of a 2-type BGWPDRE in the absence of environmental fluctuations (in this case, we use the abbreviation BGWPD). For simplicity, we further restrict ourselves to the binary branching case (following the set-up of Example 5.3 in Haccou et al. 2007), which can be thought of as a model for bacterial reproduction via binary fission and sporulation as exhibited e.g. by *Bacillus subtilis*, and summarize several standard results (that nevertheless will be proved in the appendix for the reader’s convenience). These results will then serve as a motivation and reference point for our later results involving fluctuating environments, which will also deal with more general reproductive mechanisms.

Let $p \in (0,1)$, $X_0 = 1$ and $X = (X_n)_{n \in {\mathbb {N}}_0}$ be a 1-type BGWP with offspring distribution $Q_X = p \delta _2 + (1-p)\delta _0$, where $\delta$ denotes the Dirac measure. This mechanism can be seen as a caricature of reproduction via cell division: Every individual in each generation independently either splits in two (cell division) with probability *p* or dies with probability $1-p$.

Furthermore, for $\varepsilon \in (0,p)$, $b,w \in (0,1)$ and $d \in (0, 1-w)$, let $Z_0 = (1,0)$ and $Z = (Z_n)_{n \in {\mathbb {N}}_0}$ be a 2-type BGWPD with offspring distribution, $Q_Z$, given by

$$\begin{aligned} Q_Z^1(0,0) \;&=\; 1-p + \varepsilon ,&Q_Z^2(1,0) \;&=\; w,\\ Q_Z^1(2,0) \;&=\; (p-\varepsilon ) b,&Q_Z^2(0,0) \;&=\; d,\\ Q_Z^1(0,1) \;&=\; (p-\varepsilon )(1-b),&Q_Z^2(0,1) \;&=\; 1-w-d. \end{aligned}$$

**Fig. 1:**
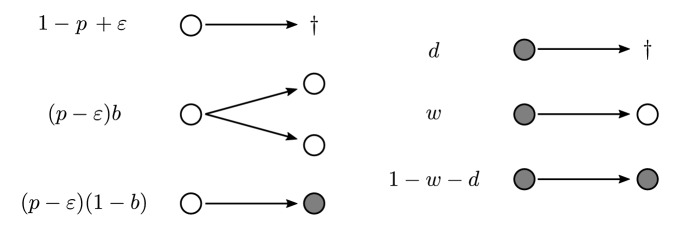
Offspring distribution of *Z* for active (white) individuals on the left and dormant (gray) individuals on the right

Figure 1 illustrates the model. The parameters can be interpreted as follows: $p-\varepsilon > 0$ is the probability with which an active individual either exhibits a reproductive or a switching event. In this case, a binary split will happen with probability *b* (reproduction), and a switching event into a dormant state (e.g. by sporulation) with probability $1-b$. Note that a ‘switch’ can be thought of as the simultaneous death of an active individual and the corresponding birth of a dormant individual. With probability $1-p+\varepsilon$, an active individual will die. Dormant individuals resuscitate (“wake up”) with probability *w*, and die with probability *d*, otherwise they stay in their dormant state (with probability $1-w-d$). Note that for $\varepsilon = 0$ and $b=1$, the active component $(Z^1_n)$ of $(Z_n)$ equals $(X_n)$ in distribution. Hence, $\varepsilon$ can be seen as a way of incorporating a reproductive trade-off that arises from the maintenance costs of the dormancy trait, delivering reduced splitting and increased death probability in comparison to the 1-type model. Additionally, note that in our model the potential to switch into dormancy also reduces the reproductive capability, since entering the seed bank is only possible during a ‘reproduction-or-switching event’ at a chance of $1-b$.

From the following more general result, which we will prove in the Appendix, we obtain a comparison of long-term survival behaviour of *X* and *Z*.

#### Proposition 2.2

Let $X = (X_n)$ be a 1-type BGWP, and $Z = (Z_n)$ a BGWPD with $X_0 = 1$ and $Z_0 = (1,0)$. Assume that the offspring distributions $Q_X$ and $Q_Z$, respectively, are of finite variance with $Q^2_Z(0,0) > 0$ and ${{\,\mathrm{{\mathbb {P}}}\,}}[Z^2_1>0] > 0$. Set $\mu _X \mathrel {\mathop :}={{\,\mathrm{{\mathbb {E}}}\,}}[X_1]$ and denote by

$$\begin{aligned}&T_{Z} \;\mathrel {\mathop :}=\; \inf \big \{n \ge 1 \;:\; Z_n = 0 \big \},&T_{X} \;\mathrel {\mathop :}=\; \inf \big \{n \ge 1 \;:\; X_n = 0 \big \}, \\&\sigma _{Z} \;\mathrel {\mathop :}=\; {{\,\mathrm{{\mathbb {P}}}\,}}\!\Big [\lim _{n \rightarrow \infty } T_Z=\infty \Big ]&\text {and}&&\sigma _{X} \;\mathrel {\mathop :}=\; {{\,\mathrm{{\mathbb {P}}}\,}}\!\Big [\lim _{n \rightarrow \infty } T_X=\infty \Big ] \end{aligned}$$

the extinction times and survival probabilities of *Z* and *X*, respectively. If

2.3 $$\begin{aligned} Q_X(k) \;\ge \; \sum _{\ell =0}^k Q_Z^1(k-\ell ,\ell ) \end{aligned}$$

for all $k \ge 1$, then the following statements hold:

1. If $$\mu _X>1$$, then $$\sigma _{Z} < \sigma _{X}$$.
2. If $\mu _X=1$, then $\sigma _{Z} = \sigma _{X}=0$ and $\, {{\,\mathrm{{\mathbb {E}}}\,}}\!\big [T_{Z}\big ] < {{\,\mathrm{{\mathbb {E}}}\,}}\!\big [T_{X}\big ] = \infty$.
3. If $\mu _X<1$, then $\sigma _{Z} = \sigma _{X} = 0$. However, $Q^2_Z$ can be chosen in such a way that $Q^2_Z(1,0)>0$, $Q^2_Z(0,0)>0$ and for some $n_0\in {\mathbb {N}}$
  2.4 $$\begin{aligned} {{\,\mathrm{{\mathbb {P}}}\,}}\!\big [T_{Z}>n \big ]&\;>\; {{\,\mathrm{{\mathbb {P}}}\,}}\!\big [T_{X} > n\big ]&\text {for all } n \ge n_0. \end{aligned}$$

Condition (2.3) ensures that the total amount of offspring of active individuals in *Z* is stochastically dominated by the amount of offspring in *X*.

Proposition 2.2 shows that—at least in the simple binary model—in the super-critical case $p>1/2$ (i.e. $\mu _X=2p>1$) maintaining a seed bank always leads to a decreased survival probability. Indeed, the reproductive trade-off, incorporated by the penalty $\varepsilon >0$, is always detrimental. The same holds for the critical regime ($p=1/2$ and $\mu _X=1$), where both processes always go extinct: Here, the expected time to extinction is even finite for the 2-type process *Z*.

However, in the sub-critical regime (3), while both processes do go extinct with probability 1, for small *w* and *d* (i.e. $Q_Z^2(1,0)$ and $Q_Z^2(0,0)$) the population with dormancy trait can be more likely to survive for extended periods of time, since by (2.4) ${{\,\mathrm{{\mathbb {P}}}\,}}[T_Z>n]>{{\,\mathrm{{\mathbb {P}}}\,}}[T_X>n]$ for $n\ge n_0$, i.e. ${{\,\mathrm{{\mathbb {P}}}\,}}[Z^1_n+Z^2_n>0]>{{\,\mathrm{{\mathbb {P}}}\,}}[X_n>0]$. This is in line with basic intuition, since for small *w* and *d* individuals spend a long time in the dormant state delaying extinction. Notably, the condition on *w* and *d* can be given explicitly, i.e. (3) holds whenever

$$\begin{aligned} 1-d \;>\; \mu _X \qquad \text {and}\qquad w \;<\; (1-d-\mu _X)\cdot \frac{\mu _X-{{\,\mathrm{{\mathbb {E}}}\,}}[Z_1^1]}{\mu _X-{{\,\mathrm{{\mathbb {E}}}\,}}[Z_1^1+Z_1^2]}. \end{aligned}$$

This suggests that the ‘prolonged survival in the sub-critical regime’ effect could lead to a fitness advantage in the presence of a random environment, fluctuating between a *healthy* (super-critical) and a *harsh* (sub-critical) scenario, even if the dormancy trait exhibits a reproductive trade-off in the healthy scenario, since dormancy could potentially compensate for this during harsh times by delaying extinction. A central goal of this article is to identify and classify scenarios in which this is indeed the case. We thus now extend our models to incorporate such a fluctuating environment.

### Branching processes with dormancy in randomly fluctuating environment

As indicated by Proposition 2.2 above, evolutionary fitness advantages resulting from a dormancy trait may be expected to manifest themselves in the presence of a random environment, where prolonged survival times may help to survive during harsh times. Here, we even expect that dormancy may turn an otherwise (overall) sub-critical process into a super-critical one, even in the presence of reproductive trade-offs. We now introduce a simple model for a fluctuating environment, randomly oscillating between two states “1” and “2”, where “1” corresponds to *healthy* and “2” to *harsh* conditions, which is identical to the environment given as an example in Dombry et al. (2011, Sections 1.1.1 and 1.1.2.).

#### Definition 2.3

(*Binary random environment*). Let $s_1, s_2 \in (0,1]$ and $s_1 \cdot s_2 < 1$. Define a discrete-time homogeneous Markov chain $(I_n)$ with values in $\{1,2\}$ via the transition matrix

$$\begin{aligned} P_I&\mathrel {\mathop :}=\; \begin{pmatrix} 1-s_1 &{}\quad s_1\\ s_2 &{}\quad 1-s_2 \end{pmatrix}, \end{aligned}$$

where $s_1$ and $s_2$ denote the *environmental switching probabilities*. Further, denote by $\pi _I=(s_2/(s_1+s_2),\ s_1/(s_1+s_2))$ the stationary distribution of $(I_n)$ and let $I_0\sim \pi _I$.

#### Remark 2.4

The initial condition for $I_0$ as well as the assertion $s_1s_2<1$ ensure that $(I_n)$ is stationary and ergodic. Hence, the process $(I_n)$ is a random environment process as defined at the beginning of this section, taking only two values.

In the remainder of this section we will not give complete definitions of any further BGWPDRE’s. We will only be interested in the mean matrices, while the exact offspring distributions will typically be irrelevant. We will also restrict our attention to the binary random environment from Definition 2.3. As indicated in (2.2), for environmental states $e \in \{1,2\}$, these matrices will be given by

$$\begin{aligned} M(e) \;=\; \begin{pmatrix} m_{\text {a}}^e &{}\quad m_{\text {d}}^e\\ w^e &{}\quad 1-w^e-d^e \end{pmatrix}, \end{aligned}$$

where $m_{\text {a}}^e$ and $m_{\text {d}}^e$ represent the average amount of active and dormant offspring of active individuals respectively, while $w^e$ and $d^e$ denote resuscitation (‘waking’) and death probabilities of dormant individuals. Our main results will concern the following three particular strategies.

#### Definition and Remark 2.5

(*Switching strategies*). The following idealized switching strategies represent important special cases that have been discussed in the literature, see e.g. Malik and Smith (2008), Lennon and Jones (2011), Dombry et al. (2011). In particular, one distinguishes between *responsive switching*, triggered by environmental conditions, and *spontaneous* or *stochastic switching*, which is assumed to happen in each individual with a certain probability, independently of the environment and the behaviour of other individuals.

- **Responsive switching**: We consider the case where individuals behave “optimally” in the sense that they invest all their resources into the production of active individuals during the healthy environmental spells (choosing $m_{\text {d}}^1=0$, $w^1=1-d^1$), while during harsh environmental conditions they invest everything into dormant offspring (choosing $m_{\text {a}}^2=w^2=0$). Hence, in this case, the offspring mean matrices are of the form
  $$\begin{aligned} M^{\mathrm {res}}(1) \;=\; \begin{pmatrix} m^1 &{}\qquad 0\\ 1-d^1 &{}\qquad 0 \end{pmatrix} \qquad \text {and}\qquad M^{\mathrm {res}}(2) \;=\; \begin{pmatrix} 0 &{}\qquad m^2\\ 0 &{}\qquad 1-d^2 \end{pmatrix} \end{aligned}$$
  for some $m^e > 0$ and $d^e < 1$.
- **Stochastic switching:** Here, the population assumes a reproductive strategy which is independent of its environmental state. This can be modeled by choosing $m_\bullet ^1 = m_\bullet > 0$ and $m_\bullet ^2 = \alpha m_\bullet$ for $\alpha \in [0,1)$ and $\bullet \in \{\text{ a }, \text{ d }\}$. That way, $m_{\text {a}}^1/m_{\text {d}}^1 = m_{\text {a}}^2/m_{\text {d}}^2$, which means that active individuals split up their resources into the production of active and dormant offspring in the same way in both environments. Then, the offspring mean matrices for $e \in \{1,2\}$ equate to
  $$\begin{aligned} M^{\mathrm {sto}}(1) \;=\; \begin{pmatrix} m_{\text {a}}&{}\qquad m_{\text {d}}\\ w^1 &{}\qquad 1-w^1-d^1 \end{pmatrix} \qquad \text {and}\qquad M^{\mathrm {sto}}(2) \;=\; \begin{pmatrix} \alpha m_{\text {a}}&{}\qquad \alpha m_{\text {d}}\\ w^2 &{}\qquad 1-w^2-d^2 \end{pmatrix}. \end{aligned}$$
  Note that $\alpha$ represents the effect of harsh environments: Since $\alpha < 1$, the expected number of offspring of active individuals is reduced in harsh environments. For $\alpha = 0$, all active individuals are killed immediately during such conditions, without producing any offspring.
- **Preliminary (or anticipatory) switching:** In this strategy, we consider individuals that invest all their resources into the production of dormant individuals during the healthy environmental spells (choosing $m_{\text {a}}^1=w^1=0$), while during harsh environmental conditions they invest everything into active offspring (choosing $m_{\text {d}}^2=0$, $w^2=1-d^2$). This can be understood as *counter-intuitive* responsive switching, always prefering the unfavorable type in reproduction, in anticipation of a coming environmental change. The corresponding offspring mean matrices, for some $m^e > 0$ and $d^e < 1$, are given by
  $$\begin{aligned} M^{\mathrm {pre}}(1) \;=\; \begin{pmatrix} 0 &{}\qquad m^1 \\ 0 &{}\qquad 1-d^1 \end{pmatrix} \qquad \text {and}\qquad M^{\mathrm {pre}}(2) \;=\; \begin{pmatrix} m^2 &{}\qquad 0 \\ 1-d^2 &{}\qquad 0 \end{pmatrix}. \end{aligned}$$

In *population genetic models* with seed bank, recently, similar types of switching have been distinguished (spontaneous vs. simultaneous switching) and these lead to topologically different limiting coalescent models describing the ancestry of a sample (see Blath et al. 2015, 2016, 2020). Here, in the presence of a random environment, we will see that the right choice of switching strategy can lead to qualitative fitness advantages, depending on the distribution of the environmental process.

Of course, less extreme variants, or even mixtures, of the above switching strategies should be interesting in practice. For example, as reported in van Vliet (2015) and Sturm and Dworkin (2015), phenotypic diversity in *Bacillus subtilis* with respect to the ‘exit from dormancy mechanisms’ seems to combine stochastic switching of some individuals with responsive switching due to environmental cues of others on the population level. However, the special form of the mean matrices in these ‘pure’ strategies makes it possible to explicitly compute or obtain suitable bounds on the corresponding maximal Lyapunov exponents, which are crucial to assess and compare the fitness of the corresponding BGWDREs, as we will see later. Interestingly, these building blocks will also allow the analysis of certain mixtures of strategies (Remark 2.21)

#### Remark 2.6

*(Comparison to multi-type branching process models considered in* Dombry et al. (2011) *and* Jost and Wang (2014)*)*. Note that the model in Definition 2.1 is closely related to a very general multi-type branching process in random environment (MBPRE) modeling phenotypic diversity (with many, even a continuum of, possible types) considered in Dombry et al. (2011), and Jost and Wang (2014), who themselves are inspired by the earlier work of Kussell and Leibler (2005). However, in their models, the authors follow a *two step procedure*, where in a first step each particle gives birth to a random amount of offspring (depending on its type and the state of the environment), and then in a second step, *independently* of that amount, the offspring particles are fitted (individually) with their new phenotypes. As the authors point out, this clearly disentangles the birth and “migration” (between phenotypes) phases. The BGWPDRE-model that we propose here is tailored to dormancy and does not disentangle these steps. This has consequences for the possible switching strategies. In fact it turns out that some of our reproductive strategies in Definition 2.5 are not covered by the framework of Dombry et al. (2011) and Jost and Wang (2014). For example, they do not cover the case that active offspring in the healthy environment can either split into two active offspring (cell division), or switch to a dormant state (e.g. by sporulation), as in the example of Sect. 2.1, since the phenotype distribution in this case would have to be allowed to depend on the number of offspring of the parent, see also Remark 3.3.

#### Remark 2.7

*(Comparison to switching strategies employed in* Malik and Smith (2008)*).* Malik and Smith in Malik and Smith (2008) consider a related, but less general switching model. Again, there are two possible environmental states, however, the bad environment here always completely prevents the reproduction of active individuals. Exact analytic expressions for the Lyapunov exponents are obtained only for the case where the environment is deterministic. In the random environment case, still some bounds are provided. It turns out that we can adapt the corresponding methods to obtain bounds for Lyapunov exponents of our models (cf. Remark 3.7).

### Asymptotic growth of BGWPDREs and Lyapunov exponents

Of particular interest is the asymptotic behaviour of the process *Z*. It is well known, cf. Kaplan (1974), that

$$\begin{aligned} {{\,\mathrm{{\mathbb {E}}}\,}}\!\big [Z_n \,\big |\, Z_0,\, \Pi = (Q_Z(e_1), Q_Z(e_2), \ldots )\big ] \;=\; Z_0 \cdot M_1 \cdot \ldots \cdot M_n. \end{aligned}$$

The study of such products of random matrices has a long and venerable history dating back to first results by Furstenberg and Kesten (1960). For stationary and ergodic sequences $(M_1, M_2, \ldots )$ of non-negative matrices satisfying ${{\,\mathrm{{\mathbb {E}}}\,}}\!\big [\log ^+ \Vert M_1\Vert \big ] < \infty$, where $\log ^+(x) \mathrel {\mathop :}=\max \{\log (x), 0\}$ and $\Vert \cdot \Vert$ is a matrix norm, Kingman (1973), see also Oseledec (1968), proved that, by means of the subadditive ergodic theorem,

2.5 $$\begin{aligned} \varphi \;\mathrel {\mathop :}=\; \lim _{n \rightarrow \infty } \frac{1}{n} \log \Vert M_1 \cdot \ldots \cdot M_n\Vert \;\in \; [-\infty , \infty ) \end{aligned}$$

exists ${{\,\mathrm{{\mathbb {P}}}\,}}$-a.s. and satisfies

$$\begin{aligned} \varphi \;=\; \lim _{n \rightarrow \infty } \frac{1}{n} {{\,\mathrm{{\mathbb {E}}}\,}}\!\Big [\log \Vert M_1 \cdot \ldots \cdot M_n\Vert \Big ]. \end{aligned}$$

Note that, by norm equivalence, $\varphi$ is independent of the chosen matrix norm. The limit, $\varphi$, is called *maximal Lyapunov exponent*.

#### Remark 2.8

*(Exact computation of Lyapunov exponents).* There are only a few cases where the maximal Lyapunov exponent can be computed explicitly. For instance, if $(M_1, M_2, \ldots )$ is a stationary and ergodic process of positive $1 \times 1$ matrices. Then, recalling that $\varrho (M)$ denotes the spectral radius of the matrix *M*, trivially $M_n = \varrho (M_n)$ for all $n \in {\mathbb {N}}$, and if ${{\,\mathrm{{\mathbb {E}}}\,}}[\log ^+ \varrho (M_1)] < \infty$ an application of Birkhoff’s ergodic theorem yields, ${{\,\mathrm{{\mathbb {P}}}\,}}$-a.s.,

2.6 $$\begin{aligned} \varphi \;=\; \lim _{n \rightarrow \infty } \frac{1}{n} \sum _{k=1}^n \log M_k \;=\; {{\,\mathrm{{\mathbb {E}}}\,}}\!\big [\log \varrho (M_1) \big ]. \end{aligned}$$

A further simple case is given by a sequence of stationary and ergodic matrices with ${{\,\mathrm{{\mathbb {E}}}\,}}[\log ^+ \Vert M_1\Vert ] < \infty$ such that the matrices $M_i$ are either mutually diagonizable, i.e. $M_iM_j = M_jM_i$ for all $i \ne j$, or upper (lower) triangular. Then, ${{\,\mathrm{{\mathbb {P}}}\,}}$-a.s.,

$$\begin{aligned} \varphi \;=\; \lim _{n \rightarrow \infty } \frac{1}{n} \log \Vert M_1 \cdot \ldots \cdot M_n\Vert \;=\; {{\,\mathrm{{\mathbb {E}}}\,}}\!\big [\log \varrho (M_1)\big ]. \end{aligned}$$

Further cases in which the maximal Lyapunov exponent can be computed explicitly are discussed in Key (1987). For the general case, where the computation of $\varphi$ is infeasible, there are various strategies for giving bounds known in the literature, see also Crisanti et al. (1993). We will discuss and employ possible methods in Sect. 3.2.

#### Remark 2.9

(Approximation of Lyapunov exponents). Under certain further assumptions on the stationary and ergodic sequence, $(M_1, M_2, \ldots )$, of non-negative matrices with ${{\,\mathrm{{\mathbb {E}}}\,}}[\log ^+ \Vert M_1\Vert ] < \infty$, Key (1990) proved that, ${{\,\mathrm{{\mathbb {P}}}\,}}$-a.s. and in mean,

$$\begin{aligned} \varphi \;=\; \lim _{n \rightarrow \infty } \frac{1}{n} \log f(M_1 \cdot \ldots \cdot M_n) \end{aligned}$$

for any one-homogeneous, non-negative, super-multiplicative function, *f*, such that ${{\,\mathrm{{\mathbb {E}}}\,}}[\log ^- f(M_1)] > -\infty$. By defining

$$\begin{aligned} \underline{\varphi }_k \;\mathrel {\mathop :}=\; \frac{1}{k} {{\,\mathrm{{\mathbb {E}}}\,}}\!\big [\log f(M_1 \cdot \ldots \cdot M_k)\big ] \qquad \text {and} \qquad {\overline{\varphi }}_k \;\mathrel {\mathop :}=\; \frac{1}{k} {{\,\mathrm{{\mathbb {E}}}\,}}\!\big [\log \Vert M_1 \cdot \ldots \cdot M_k\Vert \big ], \end{aligned}$$

then it follows from the sub-multiplicativity of $\Vert \cdot \Vert$, the super-multiplicativity of *f*, and the stationarity of the sequence $(M_1, M_2, \ldots )$ that $\underline{\varphi }_k$ increases monotonically to $\varphi$, whereas ${\overline{\varphi }}_k$ decreases monotonically to $\varphi$. Although this provides an easy way to derive upper and lower bounds on the maximal Lyapunov exponent, the computational effort increases exponentially in *k*. For i.i.d. sequences $(M_1, M_2, \ldots )$ Pollicott (2010) and Jurga and Morris (2019) established efficient approximation schemes with super-exponential convergence rates, see also Protasov and Jungers (2013) for further bounds.

#### Remark 2.10

*(Lyapunov exponent and long-term behaviour).* Notice that for a *p*-type BGWPRE, *Z*, the sequence of mean matrices, $(M_1, M_2, \ldots )$, forms a stationary and ergodic process. Thus, provided that ${{\,\mathrm{{\mathbb {E}}}\,}}\!\big [\log ^+ \Vert M(E_1)\Vert \big ] < \infty$, the corresponding maximal Lyapunov exponent, $\varphi _Z$, exists describing the asymptotic rate of growth/decay of the expected value of *Z*.

The almost sure behaviour of the process *Z* has also been studied intensively. For instance, if *Z* is a *p*-type BGWPRE such that $M_n \in (0, \infty )^{p \times p}$ for all $n \in {\mathbb {N}}$ and ${{\,\mathrm{{\mathbb {E}}}\,}}\!\big [\log ^+ \Vert M(E_1)\Vert \big ] < \infty$, then it follows from Tanny (1981, Theorem 9.10) that, for almost all realizations of the environment, $\varphi _Z < 0$ implies that *Z* becomes extinct almost surely, whereas for $\varphi _Z > 0$ there exists a positive probability that *Z* never becomes extinct. Moreover, conditioned on survival, we have that $\frac{1}{n} \log \Vert Z_n\Vert _1 \rightarrow \varphi _Z$ almost surely as $n\rightarrow \infty$. In particular, the almost sure growth of the stochastic switching model, cf. Definition 2.5-b), conditioned on non-extinction, is given by $\varphi _Z$.

Actually, Tanny established in (1981, Theorem 9.6 and Theorem 9.10) a classification theorem for more general multi-type BGWPRE with non-negative mean matrices satisfying certain regularity conditions. Notice that these conditions are not satisfied by our responsive and preliminary switching model, cf. Definition 2.5-a). However, due to the particular structure that allows a reduction of this BGWPDRE to a 1-type BGWPRE (cf. Dombry et al. 2011, Proposition 7), an analogous classification theorem can then be deduced from Tanny (1981, Theorem 9.6), see also Athreya and Karlin (1971a, 1971b).

#### Remark 2.11

(Lyapunov exponent, fitness and survival-probability). The previously mentioned features of the maximal Lyapunov exponent, describing various growth properties of population models, justifies the use of $\varphi$ as a measure of *fitness* of population models, as is common in the literature. However, there is no direct monotone relationship between $\varphi$ and the *survival probability* of the underlying population in the super-critical case, as the following example confirms: Taking the setting from Sect. 2.1, choosing *X* with parameter $p=4/7$, one can compute that $\sigma _X=2-\frac{1}{p}=0.25$ and $\varphi _X=2p\approx 1.143$. Then, for *Z* letting $p=4/5$, $\varepsilon =0$, $b=2/5$, $w=1/2$ and $d=1/25$ it holds that

$$\begin{aligned} \sigma _Z \;=\; 2 - \frac{1}{bp} + \frac{1-b}{b} \cdot \frac{w}{w+d} \;\approx \; 0.264 \;>\; \sigma _X. \end{aligned}$$

However (cf. (5.1) below), $\varphi _Z \approx 1.050 < \varphi _X$. Hence, the comparison of Lyapunov exponents of distinct population models does not necessarily provide a complete picture of the advantages of one growth strategy over the other.

This phenomenon has been studied in more detail by Jost and Wang (2014), where the authors illustrate that different optimization criteria (i.e. largest growth rate vs. smallest extinction probability) can lead to distinct optimal strategies.

We now move on to some of the main results of this paper. Indeed, the Lyapunov exponent for a BGWPDRE with responsive and preliminary switching strategy can be computed explicitly:

#### Theorem 2.12

(Lyapunov exponent of the responsive switcher). Let $(Z_n)$ be a BGWPDRE with environment process $(I_n)$ from Definition 2.3, following the *responsive* switching regime in Definition 2.5-a). Then, ${{\,\mathrm{{\mathbb {P}}}\,}}$-a.s.,

$$\begin{aligned} \varphi _Z \;=\; \frac{ s_2 \log (m^1) + s_1\log (1-d^2) + s_1 s_2\log \Big (\frac{m^2(1-d^1)}{m^1(1-d^2)}\Big )}{s_1+s_2}. \end{aligned}$$

#### Theorem 2.13

(Lyapunov exponent of the preliminary switcher). Let $(Z_n)$ be a BGWPDRE with environment process $(I_n)$ from Definition 2.3, following the *preliminary* switching regime in Definition 2.5-c). Then, ${{\,\mathrm{{\mathbb {P}}}\,}}$-a.s.,

$$\begin{aligned} \varphi _Z \;=\; \frac{ s_2 \log (1-d^1) + s_1\log (m^2) + s_1 s_2\log \Big (\frac{m^1(1-d^2)}{m^2(1-d^1)}\Big )}{s_1+s_2}. \end{aligned}$$

A proof will be provided in the next section. For the stochastic switcher we obtain the following analytic result under the additional assumption that the determinant of the mean matrices vanishes:

#### Theorem 2.14

(Lyapunov exponent of the stochastic switcher). Let $(Z_n)$ be a BGWPDRE with environment process $(I_n)$ from Definition 2.3, following the *stochastic* switching regime in Definition 2.5-b) with $\det M(1) = \det M(2) = 0.$ Then, ${{\,\mathrm{{\mathbb {P}}}\,}}$-a.s.,

2.7 $$\begin{aligned} \varphi _Z \;=\; \frac{ s_2 \log \big (m_{\text {a}}+ w^1 \frac{m_{\text {d}}}{m_{\text {a}}}\big ) + s_1 \log \big (\alpha m_{\text {a}}+ w^2 \frac{m_{\text {d}}}{m_{\text {a}}}\big ) }{s_1+s_2}. \end{aligned}$$

These results are closely related to results in Dombry et al. (2011), considering that mean matrices of determinant zero correspond to the *non-hereditary-with-sensing* case therein (cf. Sect. 3.1 for the proof and further remarks). In the *hereditary* case, i.e. the case of non-zero determinants, neither (Dombry et al. 2011) nor the article at hand obtain an explicit result for $\varphi _Z$. However, various bounds will be discussed in Sect. 3.2. We provide one of them here in a special case, for illustration:

#### Theorem 2.15

Let $(Z_n)$ be a BGWPDRE with environment process $(I_n)$ from Definition 2.3, following the *stochastic* switching regime in Definition 2.5 with $w^1 = w^2$, $d^1 = d^2$ and $\alpha \in (0,1)$. Then, ${{\,\mathrm{{\mathbb {P}}}\,}}$-a.s.,

$$\begin{aligned} \varphi _Z \;\ge \; {{\,\mathrm{{\mathbb {E}}}\,}}\!\big [ \log \big ({{\,\mathrm{\mathrm {tr}}\,}}M(I_0)-\max \{\det M(1)/m_{\text {a}},0\}\big ) \big ]. \end{aligned}$$

Note that since *w* and *d* do not depend on *e*, we get $\det M(2) = \alpha \det M(1)$. Notably, when $\det M(1)=0$, this lower bound equates to the result from Theorem 2.14.

Further bounds will be provided in the next section, where we also try to shed light on the structures of switching mechanisms that allow for the computation of analytical results and bounds. Indeed, we will distinguish the so-called ‘rank-1’-case (which is closely related to the results in Dombry et al. (2011)), allowing explicit computations, and the ‘rank-2’-case, where often only bounds can be provided. Here, we refer to the rank of mean matrices of the reproduction and switching mechanisms. Obviously, the mean matrices of the responsive and preliminary switcher in Definition 2.5 are degenerate and of rank 1, as are the mean matrices of the stochastic switcher in Theorem 2.14, due to the vanishing determinant, while the stochastic switcher of Theorem 2.15 has mean matrices of rank 2. Yet, this switching mechanism also has particular properties that will be exploited in the next section.

Before we carry out these considerations and prove the above results, we first investigate the selective advantages of the switching strategies in different environments.

### Fair comparison of BGWPDREs with different switching strategies

To decide which switching strategy of two different BGWPDREs is superior in an environment given by $(I_n)$, one needs to impose a condition that ensures that both processes “may use an equal amount of available resources”. One way to do this would be to require that both processes can produce in expectation the same amount of offspring in each generation, be it active or dormant offspring, and to assume that the death probabilities of both processes are the same in both the active and dormant states each. The processes thus can adapt to the environment only by means of their specific switching strategies while using the same amount of resources. This motivates our notion of fitness advantages under “fair comparison”. We formulate this concept in the general framework of *p*-type branching processes in random environments $(E_n)$.

#### Definition 2.16

(*Fitness advantage under fair comparison*). For $p \ge 1$ let $Z \equiv (Z_n)$ and ${\bar{Z}} \equiv ({{\bar{Z}}}_n)$ two *p*-type BGWPRE with respect to the same environmental process $(E_n)$ such that ${{\,\mathrm{{\mathbb {P}}}\,}}$-a.s. for all $1 \le t \le p$ and $n \ge 1$ their mean matrices satiesfy

2.8 $$\begin{aligned} \sum _{i=1}^p m_n^{t,i} \;=\; \sum _{i=1}^p {\overline{m}}_n^{t,i}. \end{aligned}$$

Then, if $\varphi _Z > \varphi _{{{\bar{Z}}}}$, we say that *Z* is *fitter* than ${\bar{Z}}$ at *fair comparison*. If additionally $\varphi _Z > 0 \ge \varphi _{{{\bar{Z}}}}$, we say that *Z* has a *strong* (or qualitative) fitness advantage over ${{\bar{Z}}}$ under fair comparison.

#### Remark 2.17

1. The concept of Definition 2.16 is in the same spirit as the comparison of strategies in Dombry et al. (2011), since equation (2.8) assures that for each *t*, type-*t*-individuals in both populations produce in expectation the same amount of offspring, only varying the distribution of types among offspring.
2. For BGWPDREs in environment $(I_n)$, equation (2.8) is equivalent to
  $$\begin{aligned}&(i)\quad m_{\text {a}}^e + m_{\text {d}}^e \;=\; {\overline{m}}_{\mathrm {a}}^e + {\overline{m}}_{\mathrm {d}}^e&\text {and}&(ii)\quad d^e \;=\; {\bar{d}}^e \end{aligned}$$
  for each $$e \in \{1,2\}$$.
3. To allow a comparison of a BGWPDRE to a 1-type process (i.e. without dormancy), let $(X_n)$ be a 1-type BGWPRE with environment $(I_n)$ with conditional offspring means $m_1$ and $m_2$ (referring to healthy and harsh environmental states respectively). This process can be understood as a 2-type BGWPRE process in the sense of Definition 2.1, starting in (1, 0), and having mean matrices
  $$\begin{aligned} M(e) \;=\; \begin{pmatrix} m^e &{}\quad 0\\ 1-d^e &{}\quad 0 \end{pmatrix} \end{aligned}$$
  for $e \in \{1,2\}$ and some arbitrary $d^e \in (0,1)$. Although these matrices are reducible, this makes a fair comparison feasible.
4. Note that the notion of fair comparison alone does not yet imply any kind of reproductive trade-off. However, every Lyapunov exponent or bound of such we will compute in the rest of this paper is continuous in the model parameters. This continuity and the strictness of the inequality in the definition of fitness advantages very well include the possibility of advantages under ‘disadvantageous’ comparison, including (sufficiently small but non-trivial) trade-offs.

One of the main goals of this article is to identify situations, under fair comparison, in which one switching strategy can be super-critical, whereas the other switching strategies and the process without dormancy is sub-critical. Note that this is impossible in the absence of a random environment, as pointed out in the discussion after Proposition 2.2. This is now obtained in the context of fair comparison and making use of Remark 2.17:

#### Theorem 2.18

Denote by $(I_n)$ an environment process as in Definition 2.3, by *X* a 1-type branching process as in Remark 2.17 and by $Z^{\mathrm {res}}, Z^{\mathrm {pre}}, Z^{\mathrm {sto}}$ BGWPDREs following either the responsive, preliminary or stochastic switching strategy as in Definition 2.5. Then, for either of the four processes there are non-trivial parameter regimes and environmental distributions, under which this process has a strong fitness advantage over the other three in the sense of Definition 2.16.

We prove this Theorem by means of examples of dominant strategies combining the results of Theorem 2.12, Theorem 2.13, Theorem 2.14 and (2.6) from Remark 2.8 after fitting the parameters to the regime of fair comparison.

**Fig. 2:**
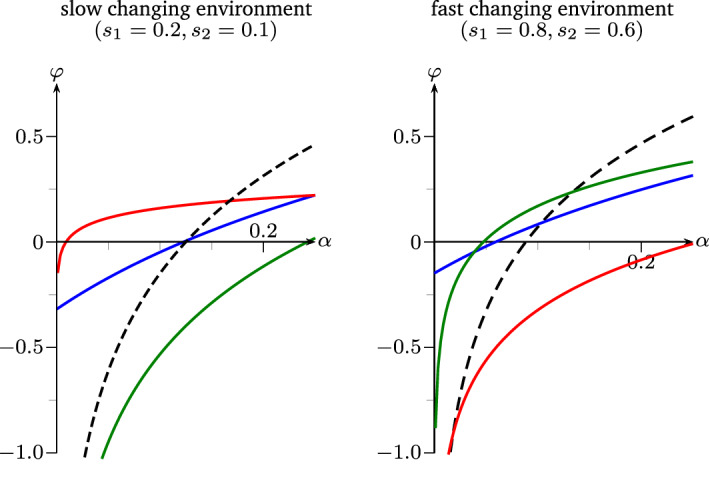
Parameter regimes of Example 2.19-(1) and -(3) respectively, Lyapunov exponents taken as functions of $\alpha$. black: $\varphi _X$, red: $\varphi _{\mathrm {res}}$, blue: $\varphi _{\mathrm {sto}}$, green: $\varphi _{\mathrm {pre}}$ (color figure online)

#### Example 2.19

*(Strong fitness advantages of seed bank switching strategies).* Let *X* be a 1-type BGWPRE as in Remark 2.17 above with $m(1) = 4$ and $m(2) = 4\alpha$, where $\alpha < 1/4$ such that *X* is sub-critical in the second environment. Further, let $Z^{\mathrm {res}}$, $Z^{\mathrm {pre}}$ and $Z^{\mathrm {sto}}$ be three BGWPDREs with mean matrices

$$\begin{aligned} M^{\mathrm {res}}(1)&\;=\; \begin{pmatrix} 4 &{}\quad 0\\ 4/5 &{}\quad 0 \end{pmatrix},&M^{\mathrm {res}}(2)&\;=\; \begin{pmatrix} 0 &{}\quad 4\alpha \\ 0 &{}\quad 4/5 \end{pmatrix}, \\ M^{\mathrm {pre}}(1)&\;=\; \begin{pmatrix} 0 &{}\quad 4\\ 0 &{}\quad 4/5 \end{pmatrix},&M^{\mathrm {pre}}(2)&\;=\; \begin{pmatrix} 4\alpha &{}\quad 0\\ 4/5 &{}\quad 0 \end{pmatrix}, \\ M^{\mathrm {sto}}(1)&\;=\; \begin{pmatrix} 2 &{}\quad 2\\ 2/5 &{}\quad 2/5 \end{pmatrix},&M^{\mathrm {sto}}(2)&\;=\; \begin{pmatrix} 2\alpha &{}\quad 2\alpha \\ 2/5 &{}\quad 2/5 \end{pmatrix}. \end{aligned}$$

Note that both the responsive and preliminary switching matrices correspond to $d^1 = d^2 = 1/5$ and the stochastic switching matrices additionally to $w^1 = w^2 = 2/5$. In particular, these processes satisfy (2.8), the condition of fair comparison. Also note that $\det M^{\mathrm {sto}}(1) = \det M^{\mathrm {sto}}(2) = 0$, such that we obtain an exact result from Theorem 2.14.

Now, only the environment-related parameters $\alpha < 1/4$ and $s_1, s_2$ are left to play with, describing the severity of harsh environments and the lengths of the environmental phases. The following cases prove Theorem 2.18:

1. For $\alpha = 1/20$, $s_1 = 2/10$ and $s_2 = 1/10$ we obtain
  $$\begin{aligned}&\varphi _X \approx -0.61< 0,&\varphi _{\mathrm {sto}} \approx -0.17< 0,&\varphi _{\mathrm {pre}} \approx -0.95 < 0&\text {but}&\\&\varphi _{\mathrm {res}} \approx 0.11 > 0. \end{aligned}$$
2. For $\alpha = 1/20$, $s_1 = 1/2$ and $s_2 = 1/2$ we get
  $$\begin{aligned}&\varphi _X \approx -0.11< 0,&\varphi _{\mathrm {res}} \approx -0.17< 0,&\varphi _{\mathrm {pre}} \approx -0.17 < 0&\text {but}&\\&\varphi _{\mathrm {sto}} \approx 0.09 > 0. \end{aligned}$$
3. Letting $\alpha = 1/20$, $s_1 = 8/10$ and $s_2 = 6/10$ implies
  $$\begin{aligned}&\varphi _X \approx -0.33< 0,&\varphi _{\mathrm {res}} \approx -0.56< 0,&\varphi _{\mathrm {sto}} \approx -0.02 < 0&\text {but}&\\&\varphi _{\mathrm {pre}} \approx 0.01 > 0. \end{aligned}$$
4. Finally, choosing $\alpha = 1/5$, $s_1 = 8/10$ and $s_2 = 3/20$ yields
  $$\begin{aligned}&\varphi _{\mathrm {res}} \approx -0.17< 0,&\varphi _{\mathrm {sto}} \approx -0.05< 0,&\varphi _{\mathrm {pre}} \approx -0.02 < 0&\text {but}&\\&\varphi _X \approx 0.03 > 0. \end{aligned}$$

**Fig. 3:**
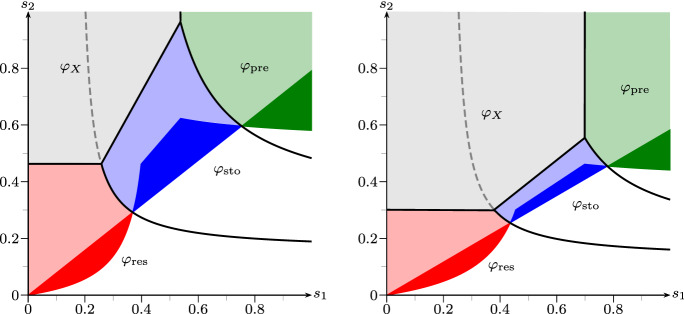
Phase diagram of fair comparison, strategies taken from Example 2.19 with fixed $\alpha =1/20$ (left) and $\alpha =1/10$ (right). The areas indicate parameter regimes where the respective strategies are advantegous. Colored regions indicate *strong* advantages, light colors indicate advantage and (not necessarily unique) supercriticality. *X* has no strong advantage in these settings. Below the colored regions (white), all strategies are subcritical. (cf. Remark 2.20) (color figure online)

#### Remark 2.20

*(Interpretation of advantageous strategies).* Figure 2 provides more insight into the behaviour of the four strategies than Example 2.19, by taking the parameter regimes (1) and (3) thereof and plotting the respective Lyapunov exponents as functions of $\alpha \in (0, 1/4)$. Furthermore, Fig. 3 shows the fitness advantage landscapes of the four models in dependence of $(s_1,s_2)$, where strong advantages are colorized.

The responsive switcher, when $m_2 \ll 1-d_2$, suffers most upon entering or exiting the harsh environment. Hence, in a scenario where environments rarely change (cf. Figs. 2 (left) and 3), responsive switching does well compared to the other strategies.

The stochastic switcher however, performs a *bet hedging* strategy, i.e. investing in dormant offspring even in good times to have better chances in worse times. This can often be very costly, but really pays off when environments change with a moderate frequency (cf. Fig. 3), especially when bad environments get very harsh, i.e. when $\alpha$ is small.

The preliminary switcher is a somewhat paradoxical form of responsive switching, since it invests under good environmental conditions all its resources in producing dormant offspring, whereas in bad environments it only produces active offspring. In the extreme case $s_1=s_2=1$, where deterministically the environment changes at every generation, it is intuitive that the preliminary switcher is optimal. Figure 3 even shows a non-trivial parameter region, in which this strategy is dominant.

Note that the 1-type process without dormancy trait will always dominate the switching strategies when $\alpha$ becomes sufficiently big—i.e. when the process gets less and less sub-critical in bad environments—as illustrated in Fig. 3 (right). In fact, in that particular parameter setting, when $\alpha \ge 1/5$, only the region of $\varphi _X$ will appear in the phase diagram, meaning that for any environmental parameters $\varphi _X \ge \max \{\varphi _{\mathrm {res}}, \varphi _{\mathrm {sto}}, \varphi _{\mathrm {pre}}\}$. This corresponds to Proposition 2.2 from the beginning of this paper, where we saw that seed bank strategies are at a disadvantage in super-critical environments.

Lastly, note that, for general values of $\alpha$, the case of iid environments—which corresponds to the line on which $s_1+s_2=1$—would not at all capture the strong advantage of responsive and preliminary switching in the settings of Fig. 3. Hence, for providing a complete understanding of the fitness landscapes, the iid case is insufficient.

**Fig. 4:**
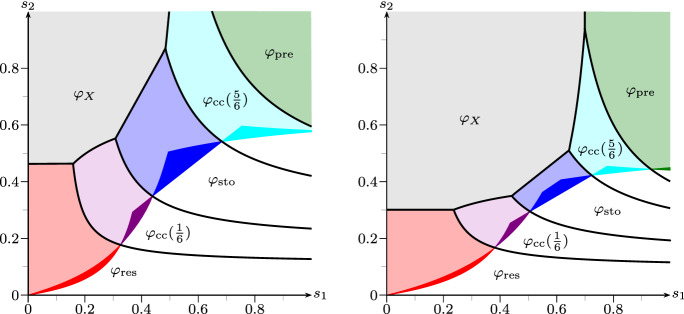
Figure 3 extended by $\varphi _{\mathrm {cc}}(1/6)$ and $\varphi _{\mathrm {cc}}(5/6)$ from Remark 2.21. Additional advantegous regions arise for the mixed strategies: purple: $\varphi _{\mathrm {cc}}(1/6)$, cyan: $\varphi _{\mathrm {cc}}(5/6)$ (color figure online)

#### Remark 2.21

*(Combining basic strategies).* The presence of phenotypic diversity regarding different switching strategies within the same Bacillus population [at least with respect to the exit strategy from dormancy, see van Vliet (2015) and Sturm and Dworkin (2015)] suggests also to investigate in mixtures of switching strategies. To model this, we let each individual choose at birth whether it behaves according to the preliminary (with probability $q(e) \in [0,1]$) or the responsive (with probability $1-q(e)$) switching mechanism. The resulting mean matrices are given by

$$\begin{aligned} M_q^{\mathrm {cc}}(e) \;\mathrel {\mathop :}=\; q(e) M^{\mathrm {pre}}(e) + (1-q(e)) M^{\mathrm {res}}(e), \end{aligned}$$

still maintaining fair comparison. Furthermore, these matrices also have determinant 0. (In fact, linear combinations of rank-1-matrices under fair comparison always retain rank 1). In particular, under fair comparison, the stochastic switcher with $\det M^{\mathrm {sto}}(1) = \det M^{\mathrm {sto}}(2) = 0$ can be represented as the convex combination of the responsive and preliminary switcher with $q(1) = m_{\text {d}}/(m_{\text {a}}+m_{\text {d}})$ and $q(2)=m_{\text {a}}/(m_{\text {a}}+m_{\text {d}})$. Hence, we can again compute their fitness explicitly, and this leads to very interesting behaviour.

Inserting the matrices of Example 2.19, we

obtain for $$q(1)=q(2)=q$$

$$\begin{aligned} M_q^{\mathrm {cc}}(1) \;=\; \begin{pmatrix} 4 (1-q) &{}\quad 4 q \\ 4(1-q)/5 &{}\quad 4q/5 \end{pmatrix} \qquad \text {and}\qquad M_q^{\mathrm {cc}}(2) \;=\; \begin{pmatrix} 4\alpha q &{}\quad 4\alpha (1-q) \\ 4q/5 &{}\quad 4(1-q)/5 \end{pmatrix}. \end{aligned}$$

Figure 4 illustrates—in comparison to Fig. 3—which influence the convex combination of the basic strategies can have. Very intuitively, the regions where $\varphi _{\mathrm {cc}}(1/6)$ and $\varphi _{\mathrm {cc}}(5/6)$ have an advantage lie between the regions of the responsive/stochastic and stochastic/preliminary strategies. Remarkably, e.g. around $(s_1,s_2)=(0.4,0.3)$ for $\alpha =1/20$ there is even a region where the convex combination of the responsive and preliminary strategy with $q=1/6$ yields a *strong* advantage possibly preventing extinction which, however, is certain for the responsive, stochastic and preliminary strategy.

This can be motivated as follows: For $s_1,s_2$ both either small or large, one of the pure strategies (responsive or preliminary) seems to be optimal. However, for moderate $s_1,s_2$ environmental variation is high and both fast switching and slow switching environmental phases might occur. Then, a combination of both strategies ensures that the worst case for neither strategy can affect the whole population. If one considers the strategy of stochastic switching as a bet-hedging strategy, then using phenotypic diversity to employ a mixture of extreme strategies might be seen as a ‘second-level’ bet-hedging strategy, now with respect to switching behaviour.

**Fig. 5:**
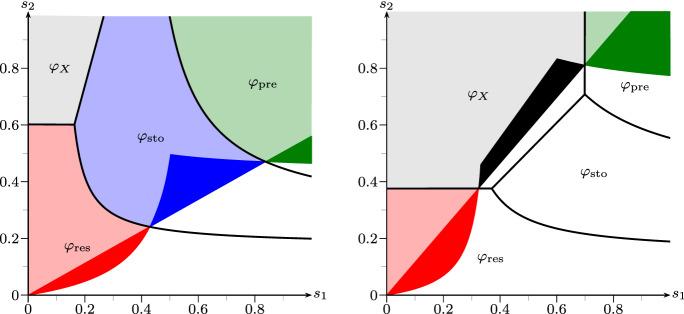
Phase diagram of $\gamma$-weighted fair comparison, strategies taken from Remark 2.22 with $\alpha = 1/20$. For $\gamma =1$ see Fig. 3 (left) (color figure online)

#### Remark 2.22

*(Refinement of fair comparison).* The notion of fair comparison implicitly assumes that the production of or conversion into dormant forms is equally costly as the production of active offspring. In many scenarios this will not be realistic. In fact, the production of inactive individuals can be both very efficient (e.g. in seed plants) as well as rather costly (e.g. sporulation of *Bacillus subtilis*, see Piggot and Hilbert 2004). Exchanging (2.8) in Definition 2.16 by

$$\begin{aligned} \sum _{i=1}^p m_n^{t,i} \gamma ^i \;=\; \sum _{i=1}^p {\overline{m}}_n^{t,i} \gamma ^i \end{aligned}$$

for some $\gamma \in (0,\infty )^p$ leads us to the notion of “$\gamma$-weighted fair comparison”. For BGWDPREs in the environment $(I_n)$ we are mainly interested in $\gamma$-weighted fair comparison with $(\gamma ^1,\gamma ^2)=(1,\gamma )$ for some $\gamma >0$. Hence, the condition above reads

2.9 $$\begin{aligned}&(i)\quad m_{\text {a}}^e + \gamma m_{\text {d}}^e \;=\; {\overline{m}}_{\text {a}}^e + \gamma {\overline{m}}_{\text {d}}^e&\text {and}&(ii)\quad d^e \;=\; {\bar{d}}^e. \end{aligned}$$

The idea behind (2.9) is to ensure that both populations still make use of the same amount of resources when producing dormant offspring becomes either less ($\gamma < 1$) or more ($\gamma >1$) resource consuming than producing active offspring. This can be seen as one particular way of incorporating a reproductive trade-off (another natural one is the introduction of the parameter $\varepsilon >0$ in the BGWPWD from Sect. 2.1). Obviously, we recover the notion of fair comparison for $\gamma =1$.

To get some intuition on the influence of $\gamma$ on the fitness under fair comparison, we provide an example: Indeed, we adjust Example 2.19 by setting

$$\begin{aligned} M^{\mathrm {res}}(2)&\;=\; \begin{pmatrix} 0 &{}\quad 4\alpha /\gamma \\ 0 &{}\quad 4/5 \end{pmatrix},&M^{\mathrm {pre}}(1)&\;=\; \begin{pmatrix} 0 &{}\quad 4/\gamma \\ 0 &{}\quad 4/5 \end{pmatrix}, \\ M^{\mathrm {sto}}(1)&\;=\; \begin{pmatrix} 4/(1+\gamma ) &{}\quad 4/(1+\gamma )\\ 2/5 &{}\quad 2/5 \end{pmatrix},&M^{\mathrm {sto}}(2)&\;=\; \begin{pmatrix} 4\alpha /(1+\gamma ) &{}\quad 4\alpha /(1+\gamma )\\ 2/5 &{}\quad 2/5 \end{pmatrix}. \end{aligned}$$

With this, the four processes from the example satisfy the condition for $\gamma$-weighted fair comparison, while we still maintain $\det M^{\mathrm {sto}}(1) = \det M^{\mathrm {sto}}(2) = 0$ to obtain an exact result from Theorem 2.14. Also, a convex combination $M_q^{\mathrm {cc}}(e)$ yields $\gamma$-weighted fair comparison, although not necessarily retaining rank 1 anymore such that $\varphi _{\mathrm {cc}}$ requires simulation.

**Fig. 6:**
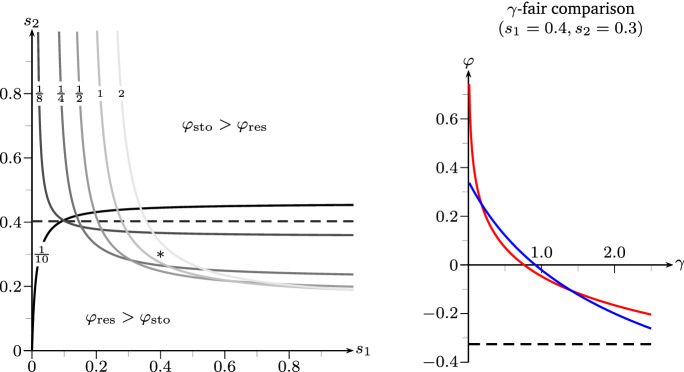
Left: Separatrices between beneficial areas of responsive versus stochastic switching with $\alpha = 1/20$ under $\gamma$-weighted fair comparison for various $\gamma$ (cf. Remark 2.22). For $\gamma =1$ compare Fig. 3 (left). $*$ marks the setting on the right. Right: Lyapunov exponents under $\gamma$-weighted fair comparison for fixed $s_1,s_2$ as functions of $\gamma$. Black: $\varphi _X$, red: $\varphi _{\mathrm {res}}$, blue: $\varphi _{\mathrm {sto}}$. Parameters given in Remark 2.22 (color figure online)

Figure 5 illustrates the influence of $\gamma$ on the phase diagram in Fig. 3 (left). Here, we see that halving the cost parameter $\gamma$ largely enhances the advantages of carrying any dormancy trait, where the advantageous region for $\varphi _{\mathrm {sto}}$ seems to increase the most. Naturally, the switching strategies will always dominate the 1-type process as $\gamma$ approaches 0, i.e. as dormant offspring become very cost-efficient. On the other hand, having a cost parameter $\gamma > 1$ shifts the landscape in such a way that the 1-type process overtakes the strong-advantage-region from the stochastic switcher.

Figure 6 (left) shows the phase diagram of stochastic versus responsive switching for various values of $\gamma$, where the separatrix in general is given by the equation

2.10 $$\begin{aligned} s_2 \;=\; \frac{ s_1 \big ( \log \big (w^2 + w^2 \tfrac{m_{\text {d}}}{m_{\text {a}}}\big ) - \log \big (\alpha m_{\text {a}}+ w^2 \tfrac{m_{\text {d}}}{m_{\text {a}}}\big ) \big ) }{ \log \big (m_{\text {a}}+ w^1 \tfrac{m_{\text {d}}}{m_{\text {a}}}\big ) - \log \big ( m_{\text {a}}+ \gamma m_{\text {d}}\big ) - s_1 \log \big (\frac{\alpha w^1}{\gamma w^2}\big ) } \end{aligned}$$

With the parameters specified above, we observe that, for $\gamma = 1/9$, the separatrix becomes a constant function with $s_2 = \log (40/29) / \log (20/9) \approx 0.4027$. Remarkably, this effect leads to parameter regimes where the fitness of the responsive switcher exceeds that of the stochastic switcher if $\gamma$ is either small *or* big, while stochastic switching wins for intermediate $\gamma$, e.g. at the point $(s_1,s_2)=(0.4,0.3)$ marked by $*$. This particular case is further depicted in Fig. 6 (right), where the respective Lyapunov exponents are plotted as functions of $\gamma$. (Note that the 1-type-fitness is constant here, since it is not influenced by the cost of dormant offspring.)

**Fig. 7:**
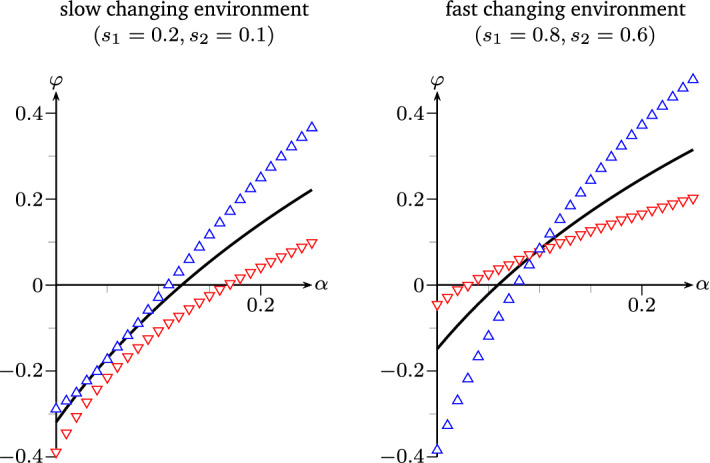
Left and right: Lyapunov exponents as functions in $\alpha$ of various stochastic switchers under fair comparison in the regimes of Example 2.19-(1) and -(3) respectively. Line: $\varphi _{\mathrm {sto}}$, ${\Delta },{\nabla }$: simulated values of $\varphi$ for strategies defined by ${M_\Delta ^{\mathrm {sto}}},{M_\nabla ^{\mathrm {sto}}}$ from Remark 2.23

#### Remark 2.23

*(Non-zero determinant case for mean matrices).* Rather than combining the pure strategies, one can also compare different stochastic switching strategies under fair comparison, e.g. by choosing mean matrices of non-zero determinant, as illustrated in Fig. 7. Here, we add to the setting of Example 2.19 two further stochastic switchers with matrices

$$\begin{aligned} M^{\mathrm {sto}}_{\Delta }(e) \;=\; \begin{pmatrix} 13\alpha ^{e-1}/4 &{}\quad 3\alpha ^{e-1}/4 \\ 2/5 &{}\quad 2/5 \end{pmatrix} \quad \text {and}\quad M^{\mathrm {sto}}_{\nabla }(e) \;=\; \begin{pmatrix} 3\alpha ^{e-1}/4 &{}\quad 13\alpha ^{e-1}/4 \\ 2/5 &{}\quad 2/5 \end{pmatrix} \end{aligned}$$

for $e \in \{1,2\}$. These satisfy the conditions of fair comparison to the processes in Example 2.19 while $\det M^{\mathrm {sto}}_{\Delta }(e) = \alpha ^{e-1} > 0$ and $\det M^{\mathrm {sto}}_{\nabla }(e) = -\alpha ^{e-1} < 0$.

In contrast to the stochastic switcher given by $M^{\mathrm {sto}}$ in Example 2.19, the $\Delta$-matrices describe a strategy that focuses more on the production of active offspring, making better use of good times. Note that a positive determinant could also come from a decrease of the resuscitation rate $w^e$, increasing the chance of enduring long-lasting harsh times. Both effects increase the fitness in less frequently changing environments, Fig. 7 (left).

The $\nabla$-matrices, however, describe a population that mostly produces dormant offspring, making for a strategy that prevails in frequently changing and sufficiently harsh environments as seen in Fig. 7 (right).

**Fig. 8:**
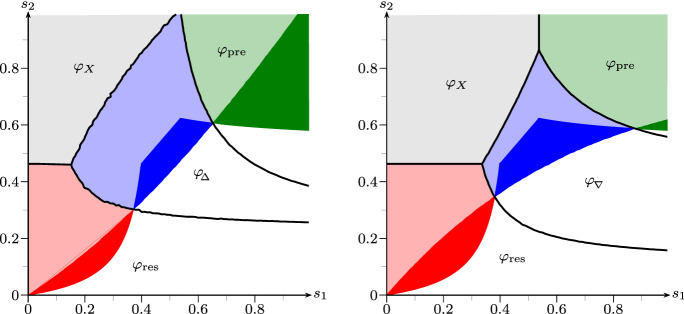
Same as Fig. 3, but $\varphi _{\mathrm {sto}}$ replaced by strategies taken from Remark 2.23 (color figure online)

Figure 8 illustrates the influence of the determinant on the phase diagram: In comparison to Fig. 3 (left), roughly, the $\Delta$-strategy with positive determinant wins ground on the left half, but loses ground on the right half of the diagram, while the $\nabla$-strategy with negative determinant generates the opposite effect.

Of course, the above examples invite a much larger and systematic study of parameter ranges and switching strategies, but we think that this goes beyond the scope of the present paper, with its focus on mathematical methods.

## Technical results for rank-1 and rank-2 switching strategies

In this section, we provide theoretical results for the explicit computation and bounds for maximal Lyapunov exponents related to switching strategies in BGWPDRE. We prove Theorems 2.12, 2.13, 2.14 (rank-1 case) and 2.15 (rank-2 case) and provide bounds in the rank-2 case and a short literature review.

### Rank-1-matrices and exact results

Consider a stationary and ergodic sequence $(M_1, M_2, \ldots )$ of non-negative $p \times p$ matrices such that, for any $n \in {\mathbb {N}}$, the rank of $M_n$ is equal to one. Hence, for any $n \in {\mathbb {N}}$, we can find column vectors $\ell _n, r_n \in [0, \infty )^p$ such that $M_n = \ell _n \cdot r_n^\top$ and the sequence $((\ell _n,r_n))_{n\ge 1}$ is stationary. Note that in this case, only one eigenvalue of $M_n$ is non-zero and, as a consequence, $\varrho (M_n) = {{\,\mathrm{\mathrm {tr}}\,}}M_n$.

#### Lemma 3.1

Let $(M_1, M_2, \ldots )$ be a stationary and ergodic sequence of non-negative $p \times p$ matrices with $M_n = \ell _n \cdot r_n^\top$ for any $n \in {\mathbb {N}}$. Suppose that ${{\,\mathrm{{\mathbb {E}}}\,}}\!\big [\log ^+\Vert \ell _1\Vert \big ] < \infty$ and ${{\,\mathrm{{\mathbb {E}}}\,}}\!\big [ \log ^+ \Vert r_1\Vert \big ] < \infty$. Then, ${{\,\mathrm{{\mathbb {P}}}\,}}$-a.s. and in mean,

3.1 $$\begin{aligned} \varphi \;=\; \lim _{n \rightarrow \infty } \frac{1}{n} \log \Vert M_1 \cdot \ldots \cdot M_n\Vert \;=\; {{\,\mathrm{{\mathbb {E}}}\,}}\!\big [ \log \, \langle r_1, \ell _2 \rangle \big ]. \end{aligned}$$

#### Proof

First, note that both ${{\,\mathrm{{\mathbb {E}}}\,}}\!\big [\log ^+ \langle r_1, \ell _2 \rangle \big ] < \infty$ and ${{\,\mathrm{{\mathbb {E}}}\,}}\!\big [\log ^+ \Vert M_1\Vert \big ] < \infty$. The latter ensures that the maximal Lyapunov exponent, $\varphi$, exists ${{\,\mathrm{{\mathbb {P}}}\,}}$-a.s. and in mean. Thus it remains to show that $\varphi$ is equal to the expression on the right-hand side of (3.1).

In order to apply (Kingman 1973, Theorem 1) which ensures that $\varphi$ is finite, we first assume

3.2 $$\begin{aligned} {{\,\mathrm{{\mathbb {E}}}\,}}\!\big [\log \Vert M_1 \cdot \ldots \cdot M_n\Vert \big ] \ge -A n \qquad \text {for some }A \in [0, \infty )\text { and all }n \in {\mathbb {N}}. \end{aligned}$$

This implies that $\log \, \langle r_1, \ell _2 \rangle \in L^1({{\,\mathrm{{\mathbb {P}}}\,}})$, $\log \Vert \ell _1\Vert \in L^1({{\,\mathrm{{\mathbb {P}}}\,}})$ and $\log \Vert r_1\Vert \in L^1({{\,\mathrm{{\mathbb {P}}}\,}})$. In particular, ${{\,\mathrm{{\mathbb {P}}}\,}}$-a.s., $\langle r_n, \ell _{n+1} \rangle > 0$ for all $n \in {\mathbb {N}}$. Since

3.3 $$\begin{aligned} \frac{1}{n} \log \Vert M_1 \cdot \ldots \cdot M_n\Vert \;=\; \frac{1}{n} \sum _{i=1}^{n-1} \log \, \langle r_i, \ell _{i+1} \rangle \,+\, \frac{1}{n} \log \Vert \ell _1 \cdot r_n^\top \Vert , \end{aligned}$$

we immediately deduce from Birkhoff’s ergodic theorem that the first term on the right-hand side of (3.3) converges, ${{\,\mathrm{{\mathbb {P}}}\,}}$-a.s. and in $L^1({{\,\mathrm{{\mathbb {P}}}\,}})$, to ${{\,\mathrm{{\mathbb {E}}}\,}}\!\big [\log \langle \ell _1, r_2 \rangle \big ]$ as $n \rightarrow \infty$. Since $\sup _{n \in {\mathbb {N}}} {{\,\mathrm{{\mathbb {E}}}\,}}\!\big [|\log \Vert \ell _1 \cdot r_n^\top \Vert |\big ] < \infty$, it follows that $\lim _{n \rightarrow \infty } \frac{1}{n} {{\,\mathrm{{\mathbb {E}}}\,}}\!\big [| \log \Vert \ell _1 \cdot r_n^\top \Vert |\big ] = 0$, and (3.1) holds in mean. Moreover, for any $\varepsilon > 0$

$$\begin{aligned} \sum _{n=1}^{\infty } {{\,\mathrm{{\mathbb {P}}}\,}}\!\big [|\log \Vert \ell _1 \cdot r_n^\top \Vert | \ge \varepsilon n\big ] \;\le \; \frac{2 C}{\varepsilon } \Big ( {{\,\mathrm{{\mathbb {E}}}\,}}\!\big [|\log \Vert \ell _1\Vert |\big ] \,+\, {{\,\mathrm{{\mathbb {E}}}\,}}\!\big [|\log \Vert r_1\Vert |\big ] \Big ) \;<\; \infty , \end{aligned}$$

where the constant $C \ge 1$ appearing in the computation above results from the comparison of equivalent matrix norms. Thus, by using the Borel–Cantelli Lemma we conclude that $\lim _{n \rightarrow \infty } \log \Vert \ell _1 \cdot r_n^\top \Vert / n = 0$ ${{\,\mathrm{{\mathbb {P}}}\,}}$-a.s., and (3.1) follows.

However, if the additional assumption in (3.2) is violated then we conclude that ${{\,\mathrm{{\mathbb {E}}}\,}}\!\big [\log \langle r_1, \ell _2 \rangle \big ] = -\infty$. Thus, by Kingman (1973, Theorem 2), the maximal Lyapunov exponent, $\varphi$, as well as the limit of the sum on the right-hand side of (3.3) exists with probability one, and $\varphi = -\infty$. Using that $\sup _{n \in {\mathbb {N}}} {{\,\mathrm{{\mathbb {E}}}\,}}\!\big [\log ^+ \Vert \ell _1 \cdot r_n^\top \Vert \big ] < \infty$ concludes the proof. $\square$

#### Corollary 3.2

Let *Z* be a *p*-type BGWPRE in environment $(I_n)$ given in Definition 2.3. Suppose that ${{\,\mathrm{\mathrm {rk}}\,}}M(e) = 1$, ${{\,\mathrm{\mathrm {tr}}\,}}M(e) > 0$ for any $e \in \{1,2\}$, and ${{\,\mathrm{\mathrm {tr}}\,}}( M(1) \cdot M(2) ) > 0$. Then, ${{\,\mathrm{{\mathbb {P}}}\,}}$-a.s.,

$$\begin{aligned} \varphi _Z \;=\; \tfrac{s_2}{s_1+s_2}\log ({{\,\mathrm{\mathrm {tr}}\,}}M(1)) + \tfrac{s_1}{s_1+s_2}\log ({{\,\mathrm{\mathrm {tr}}\,}}M(2)) + \tfrac{s_1s_2}{s_1+s_2} \log \left( \frac{{{\,\mathrm{\mathrm {tr}}\,}}(M(1)M(2))}{{{\,\mathrm{\mathrm {tr}}\,}}M(1){{\,\mathrm{\mathrm {tr}}\,}}M(2)}\right) . \end{aligned}$$

#### Proof

By Lemma 3.1, it holds that, ${{\,\mathrm{{\mathbb {P}}}\,}}$-a.s.,

$$\begin{aligned} \varphi _Z \;=\; {{\,\mathrm{{\mathbb {E}}}\,}}\!\big [ \log \, \langle r(I_0), \ell (I_1) \rangle \big ] \;=\; \sum _{i,j \in \{1,2\}} \pi _I(i)\, P_I(i,j) \log \,\langle r(i), \ell (j) \rangle . \end{aligned}$$

By using that $\langle \ell (e), r(e)\rangle = {{\,\mathrm{\mathrm {tr}}\,}}M(e)$ for any $e \in \{1,2\}$, $\langle r(1), \ell (2)\rangle \langle r(2), \ell (1)\rangle = {{\,\mathrm{\mathrm {tr}}\,}}(M(1) \cdot M(2))$ and $\pi _I(1) P_I(1,2) = \pi _I(2) P_I(2,1)$, the assertion follows. $\square$

#### Proof of Theorem 2.12, 2.13 and 2.14

This follows directly from Corollary 3.2. $\square$

#### Remark 3.3

*(Connection to* Dombry et al. 2011*).*

1. Similarly to Dombry et al. (2011, Propositions 1 and 7), the responsive switcher can be regarded as a 1-type BGWPRE process in a more complex random environment, here given by $((I_n,I_{n+1}))_n$ with corresponding offspring means $m_{1,i} = m_i$ and $m_{2,i} = 1-d_i$ for $i \in \{1,2\}$. With this, Theorem 2.12 follows by applying the Ergodic Theorem.
2. For a given fixed mean offspring per type and environment, say $(m_t(e))_{1\le t \le p}$ for $e \in \{1,2\}$, consider a collection of distributions of offspring types—say $\nu _t(e) \in {\mathbb {R}}_{\ge 0}^p$ for $1 \le t \le p$, $e \in \{1,2\}$—as reproduction strategy. In Dombry et al. (2011) the maximal Lyapunov exponent is only computed explicitly in the so-called *non-hereditary* case, that is, when the distributions of offspring types do not depend on the parent type, $\nu _t(e) = \nu (e)$. Regarding $m(e) = (m_t(e))_t$ and $\nu (e)$ as column vectors in ${\mathbb {R}}_{\ge 0}^p$, the corresponding mean matrices are $M(e) = m(e)\cdot \nu (e)^\top$ and thus, of rank 1. Hence, the case in which Dombry et al. (2011) obtain exact results for $\varphi _Z$ aligns with the case where we do. A natural generalization is to give type-*t*-individuals an offspring type distribution depending on *e* as a convex combination of two distributions, say $\nu (e)$ and $\mu (e)$. This provides a simple example of the *hereditary* case and produces mean matrices of rank at most 2.

### Rank-2-matrices and bounds

Let $(M_1, M_2, \ldots )$ a stationary and ergodic sequence of non-negative $p \times p$ matrices such that, for any $n \in {\mathbb {N}}$, the rank of $M_n$ is at most two, i.e. there are column vectors $\ell _n^i, r_n^i \in [0, \infty )^p$, $i \in \{1,2\}$ such that

$$\begin{aligned} M_n \;=\; \sum _{i=1}^2 \ell _n^i \cdot (r_n^i)^\top \end{aligned}$$

for any $i \in {\mathbb {N}}$. Further, for any $i \in {\mathbb {N}}$, we denote by $A_{n,n+1}$ a non-negative $2 \times 2$ matrix that is defined by

3.4 $$\begin{aligned} A_{n,n+1} \;\mathrel {\mathop :}=\; \begin{pmatrix} \langle r_n^1, \ell _{n+1}^1 \rangle &{}\quad \langle r_n^1, \ell _{n+1}^2 \rangle \\ \langle r_n^2, \ell _{n+1}^1 \rangle &{}\quad \langle r_n^2, \ell _{n+1}^2 \rangle \end{pmatrix}. \end{aligned}$$

Note that the sequence $(A_{1,2}, A_{2,3}, \ldots )$ is as well stationary and ergodic.

#### Remark 3.4

There are several ways to decompose a non-negative $2\times 2$-matrix into the sum of two products of non-negative vectors, e.g. for any $a,b,c,d\ge 0$ and $ab>0$ it holds

3.5 $$\begin{aligned} M \;\mathrel {\mathop :}=\; \begin{pmatrix} a&{}\qquad b\\ c&{}\qquad d \end{pmatrix}&\;=\; \begin{pmatrix} 1 \\ 0 \end{pmatrix} \cdot \begin{pmatrix} a&b \end{pmatrix} + \begin{pmatrix} 0 \\ 1 \end{pmatrix} \cdot \begin{pmatrix} c&d \end{pmatrix} \end{aligned}$$

3.6 $$\begin{aligned}&\;=\; \begin{pmatrix} a \\ c \end{pmatrix} \cdot \begin{pmatrix} 1&0 \end{pmatrix} + \begin{pmatrix} b \\ d \end{pmatrix} \cdot \begin{pmatrix} 0&1 \end{pmatrix} \end{aligned}$$

3.7 $$\begin{aligned}&\;=\; \begin{pmatrix} a \\ c \end{pmatrix} \cdot \begin{pmatrix} 1&\frac{b}{a} \end{pmatrix} + \begin{pmatrix} 0 \\ 1 \end{pmatrix} \cdot \begin{pmatrix} 0&\frac{\det M}{a} \end{pmatrix} \end{aligned}$$

3.8 $$\begin{aligned}&\;=\; \begin{pmatrix} b \\ d \end{pmatrix} \cdot \begin{pmatrix} \tfrac{a}{b}&\ 1 \end{pmatrix} + \begin{pmatrix} 0 \\ 1 \end{pmatrix} \cdot \begin{pmatrix} \frac{-\det M}{b}&0 \end{pmatrix}, \end{aligned}$$

where the entries in (3.5) and (3.6) are always non-negative, in (3.7) they are non-negative if $\det M\ge 0$ and in the last if $\det M\le 0$. Notably, (3.6) corresponds to writing *M*(*e*) as convex combination of responsive and preliminary switchers as indicated in Remark 2.21.

#### Lemma 3.5

Let $(M_1, M_2, \ldots ).$ be a stationary and ergodic sequence of non-negative $p \times p$ matrices with $M_n = \sum _{i=1}^2 \ell _n^i \cdot (r_n^i)^\top$ for any $n \in {\mathbb {N}}$ and $\log \Vert \ell _1^i\Vert , \log \Vert r_1^i\Vert \in L^1({{\,\mathrm{{\mathbb {P}}}\,}})$ for any $i \in \{1,2\}$. Then, ${{\,\mathrm{{\mathbb {P}}}\,}}$-a.s. and in mean,

3.9 $$\begin{aligned} \varphi \;=\; \lim _{n \rightarrow \infty } \frac{1}{n} \log \Vert M_1 \cdot \ldots \cdot M_n\Vert \;=\; \lim _{n \rightarrow \infty } \frac{1}{n} \log \Vert A_{1,2} \cdot \ldots \cdot A_{n-1,n}\Vert . \end{aligned}$$

#### Proof

First, by an elementary computation, we get that ${{\,\mathrm{{\mathbb {E}}}\,}}\!\big [\log ^+ \Vert M_1\Vert \big ] < \infty$ and ${{\,\mathrm{{\mathbb {E}}}\,}}\!\big [\log ^+ \Vert A_{1,2}\Vert \big ] < \infty$. Thus, Kingman (1973, Theorem 6) implies that

$$\begin{aligned} \lim _{n \rightarrow \infty } \frac{1}{n} \log \Vert M_1 \cdot \ldots \cdot M_n\Vert \qquad \text {and} \qquad \lim _{n \rightarrow \infty } \frac{1}{n} \log \Vert A_{1,2} \cdot \ldots \cdot A_{n-1,n}\Vert \end{aligned}$$

exist ${{\,\mathrm{{\mathbb {P}}}\,}}$-a.s. and in mean. Thus, we are left with showing that both limits coincide. Recall that the limit does not depend on the chosen matrix norm. Choosing $\Vert B\Vert \mathrel {\mathop :}=\sum _{i,j=1}^p |B^{i,j}|$, we obtain

$$\begin{aligned} \Vert M_1 \cdot \ldots \cdot M_n \Vert \;=\; \sum _{i,j=1}^2 \big (A_{1,2} \cdot \ldots \cdot A_{n-1,n}\big )^{i,j}\, \Vert \ell _1^i\Vert _1\, \Vert r_n^j\Vert _1. \end{aligned}$$

By setting $R_n \mathrel {\mathop :}=\sum _{i=1}^2 (|\log \Vert \ell _1^i\Vert _1| + |\log \Vert r_n^i\Vert _1 |)$ for any $n \in {\mathbb {N}}$, it follows that

$$\begin{aligned} -\frac{1}{n} R_n \;\le \; \frac{1}{n} \log \Vert M_1 \cdot \ldots \cdot M_n\Vert \,-\, \frac{1}{n} \log \Vert A_{1,2} \cdot \ldots \cdot A_{n-1,n} \Vert \;\le \; \frac{1}{n} R_n. \end{aligned}$$

Thus, by using the same argument as in the proof of Lemma 3.1, we obtain that, ${{\,\mathrm{{\mathbb {P}}}\,}}$-a.s. and in mean, $\lim _{n \rightarrow \infty } \frac{1}{n} R_n = 0$, which concludes the proof. $\square$

Next, we focus on establishing various bounds for the maximal Lyapunov exponent for the resulting product of $2 \times 2$ matrices.

#### Proposition 3.6

Let $(I_n)$ be a stationary and ergodic Markov chain with values in $\Omega ' = \{1,2\}$ as given in Definition 2.3, and $A\!: \Omega ' \times \Omega ' \rightarrow [0, \infty )^{2 \times 2}$ such that ${{\,\mathrm{{\mathbb {E}}}\,}}[|\log \Vert A(I_0, I_1)\Vert |] < \infty$. Denoting $A_{n,n+1}=A(I_n,I_{n+1})$, the following holds:

- For $\lambda \!: \Omega ' \times \Omega ' \rightarrow (0, \infty )$, let $A^*(i,j)=A(i,j)/\lambda (i,j)$ for $i,j\in \Omega '$. Then, ${{\,\mathrm{{\mathbb {P}}}\,}}$-a.s. and in mean,
  $$\begin{aligned} \lim _{n \rightarrow \infty } \frac{1}{n} \log \big \Vert A_{1,2} \cdot \ldots \cdot A_{n-1,n}\big \Vert \;\le \; {{\,\mathrm{{\mathbb {E}}}\,}}[\log \lambda (I_0,I_1)] \,+\, \log \varrho ({\widehat{A}}^*), \end{aligned}$$
  where $\varrho ({\widehat{A}}^*)$ denotes the spectral radius of the $(4 \times 4)$-matrix
  $$\begin{aligned} {\widehat{A}}^* \;\mathrel {\mathop :}=\; \begin{pmatrix} (1-s_1) A^*(1,1) &{}\qquad s_1 A(1,2)^* \\ s_2 A(2,1)^* &{}\qquad (1-s_2) A(2,2)^* \end{pmatrix}. \end{aligned}$$
- For $n\ge 1$ denote by ${\mathcal {D}}_n$ the set of probability density functions on $\{1,2\}^n$. Then, ${{\,\mathrm{{\mathbb {P}}}\,}}$-a.s. and in mean,
  $$\begin{aligned} \lim _{n \rightarrow \infty } \frac{1}{n} \log \big \Vert A_{1,2} \cdot \ldots \cdot A_{n-1,n}\big \Vert \;\ge \; \limsup _{n\rightarrow \infty }\sup _{\nu \in {\mathcal {D}}_n} \frac{1}{n} \bigg ( \sum _{k=1}^{n-1} {\mathbf {E}}_\nu [X_k] + H(\nu ) \bigg ), \end{aligned}$$
  where $X_k = \log \big (A(I_k,I_{k+1})^{\alpha _k,\alpha _{k+1}}\big )$, ${\mathbf {E}}_\nu$ denotes integration by $\alpha \in \{1,2\}^n$ with respect to $\nu$ and $H(\nu )$ the entropy of $\nu$, i.e.
  $$\begin{aligned} H(\nu ) \;=\; -\sum _{\alpha \in \{1,2\}^n} \nu (\alpha )\log \nu (\alpha ). \end{aligned}$$

#### Proof

(*a*) First, by the ergodic theorem, letting $A^*_{n,n+1}=A^*(I_n,I_{n+1})$, we have that, ${{\,\mathrm{{\mathbb {P}}}\,}}$-a.s. and in mean,

$$\begin{aligned}&\lim _{n \rightarrow \infty } \frac{1}{n} \log \big \Vert A_{1,2} \cdot \ldots \cdot A_{n-1,n}\big \Vert \\&\quad =\; {{\,\mathrm{{\mathbb {E}}}\,}}\!\big [\log \lambda (I_0, I_1)\big ] \,+\, \lim _{n \rightarrow \infty } \frac{1}{n} {{\,\mathrm{{\mathbb {E}}}\,}}\!\Big [\log \big \Vert A^*_{1,2} \cdot \ldots \cdot A^*_{n-1,n}\big \Vert \Big ]. \end{aligned}$$

Moreover, it is well known that an upper bound for the maximal Lyapunov exponent of the stationary and ergodic sequence $(A^*_{1,2}, A^*_{2,3}, \ldots )$ follows immediately from Jensen’s inequality. Indeed, using the matrix norm $\Vert B\Vert = \sum _{i,j} |B^{i,j}|$, $B \in {\mathbb {R}}^{2 \times 2}$,

$$\begin{aligned} {{\,\mathrm{{\mathbb {E}}}\,}}\!\Big [\log \big \Vert A^*_{1,2} \cdot \ldots \cdot A^*_{n-1,n}\big \Vert \Big ]&\;\le \; \log {{\,\mathrm{{\mathbb {E}}}\,}}\!\Big [\big \Vert A^*_{1,2} \cdot \ldots \cdot A^*_{n-1,n}\big \Vert \Big ]\\&\;=\; \log \big ( (\pi _I \otimes {\mathbf {1}}_2)^\top ({\widehat{A}}^*)^{n-1} ({\mathbf {1}}_4) \big ) \;\le \; \log \Vert ({\widehat{A}}^*)^{n-1}\Vert , \end{aligned}$$

where ${\mathbf {1}}_k \mathrel {\mathop :}=(1, \ldots , 1)^\top \in {\mathbb {R}}^k$. Hence, the assertion follows from Horn and Johnson (1990, Corollary 5.6.14).

(*b*) Note that, for any $\nu \in {\mathcal {D}}_n$, $\Vert A_{1,2} \cdot \ldots \cdot A_{n-1,n}\Vert$ equates to

$$\begin{aligned} \sum _{\alpha \in \{1,2\}^n} \prod _{k=1}^{n-1} A_{k, k+1}^{\alpha _k, \alpha _{k+1}} \;\ge \; \sum _{\alpha :\,\nu (\alpha )>0} \nu (\alpha ) \exp \!\bigg ( \sum _{k=1}^{n-1}\log \big (A_{k,k+1}^{\alpha _k,\alpha _{k+1}}\big ) - \log \nu (\alpha ) \bigg ). \end{aligned}$$

The result follows from Jensen’s inequality, taking supremum and limit superior. Note that the right-hand side converges almost surely and hence in mean by monotone convergence. $\square$

#### Proof of Theorem 2.15

Since *w* and *d* do not depend on *e*, it holds $\det M(2) = \alpha \det M(1)$. Notably, when $\det M(1)=0$, this lower bound equates to the result from Theorem 2.14. Hence, in what follows we assume $\det M(1)\ne 0$.

For $\det M(1) > 0$, using representation (3.7) we obtain

$$\begin{aligned} A(i,j) \;=\; \begin{pmatrix} \alpha ^{j-1}m_{\text {a}}+\frac{wm_{\text {d}}}{m_{\text {a}}} &{}\qquad \frac{m_{\text {d}}}{m_{\text {a}}} \\ \frac{w\det M(1)}{m_{\text {a}}} &{}\qquad \frac{\det M(1)}{m_{\text {a}}} \end{pmatrix}, \end{aligned}$$

where $A(i,j)_{1,1}={{\,\mathrm{\mathrm {tr}}\,}}M(j)-\frac{\det M(1)}{m_{\text {a}}}$. Otherwise, if $\det M(1)<0$ and we use (3.8),

$$\begin{aligned} A(i,j) \;=\; \begin{pmatrix} {{\,\mathrm{\mathrm {tr}}\,}}M(j) &{}\qquad 1\\ -\det M(j) &{}\qquad 0 \end{pmatrix}. \end{aligned}$$

Hence, in both cases it holds $A(i,j)_{1,1}={{\,\mathrm{\mathrm {tr}}\,}}M(j)-(\det M(1)/m_{\text {a}})^+$ and Proposition 3.6-(b) concludes the proof by considering $\nu _n=\delta _{\{1\}^n}\in {\mathcal {D}}_n$ and applying the Ergodic Theorem. $\square$

#### Remark 3.7

*(Further bounds on the maximal Lyapunov exponent).* Let us consider the stochastic switching model with mean matrices *M*(1) and *M*(2). In view of Remark 2.9 any sub-multiplicative function $\Vert \cdot \Vert \!: {\mathbb {R}}_{\ge }^{2 \times 2} \rightarrow (0, \infty )$ yields that $\varphi _Z \le {{\,\mathrm{{\mathbb {E}}}\,}}[\log \Vert M(I_0)\Vert ]$. Likewise, for any super-multiplicative function $f\!: {\mathbb {R}}_{\ge }^{2 \times 2} \rightarrow (0, \infty )$ we obtain that $\varphi _Z \ge {{\,\mathrm{{\mathbb {E}}}\,}}[\log f(M(I_0))]$. Examples of super-multiplicative functions are the minimal column and row sums, respectively, any diagonal element, or the permanent of a matrix *A*.

For a slightly improved upper bound note that for any sub-multiplicative matrix norm $\Vert \cdot \Vert$

$$\begin{aligned} \Big \Vert \prod _{k=1}^nM_k\Big \Vert \;\le \; \prod _{k=1}^n\Vert M_k\Vert \cdot \prod _{k=1}^{n-1}\Big ( \frac{\Vert M(1)M(2)\Vert }{\Vert M(1)\Vert \Vert M(2)\Vert } \Big )^{\mathbbm{1}_{I_k=1,I_{k+1}=2}}, \end{aligned}$$

which takes into account the effects of one type of environmental change. Hence, we obtain that $\varphi _Z \le {{\,\mathrm{{\mathbb {E}}}\,}}[\log \Vert M(I_0)\Vert ] + \Psi$, where

$$\begin{aligned} \Psi \;=\; \frac{s_1 s_2}{s_1+s_2} \log \Big ( \frac{\min \{\Vert M(1)M(2)\Vert ,\Vert M(2)M(1)\Vert \}}{\Vert M(1)\Vert \Vert M(2)\Vert } \Big ) \;\le \; 0. \end{aligned}$$

**Fig. 9:**
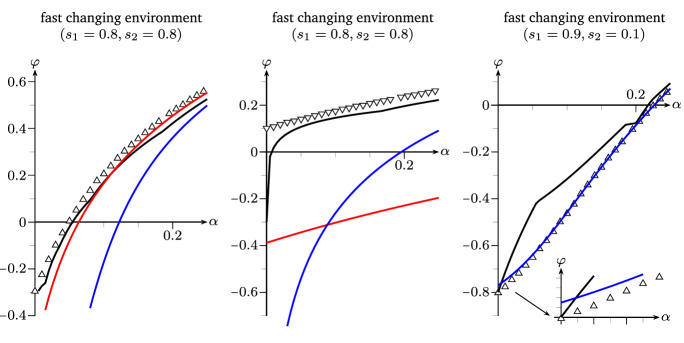
Left and mid: Comparison of lower bounds of $\Delta$- and $\nabla$-strategy resp. in setting of Fig. 7 (right)—red: Theorem 2.15, black: Corollary 3.8 maximized over 1000 random choices of the $\mu$-parameters, blue: (Hautphenne and Latouche 2016, Theorem 3), $\Delta$ and $\nabla$: approximation via simulation. On the right: Comparison of upper bounds—black: improved norm bound from Remark 3.7 with respect to $\Vert \cdot \Vert _1$, blue: (Hautphenne and Latouche 2016, Theorem 2), $\Delta$: approximation via simulation (color figure online)

As one can see in Fig. 9 (right), for small $\alpha$ in some cases this can give a better upper bound than the one from Hautphenne and Latouche (2016).

A more evolved approach is to choose a sequence $(\nu _n)$ such that $(\alpha _k)_k$ can be interpreted as path of a Markov chain. Combining this ansatz with Markov chain limit results leads to

#### Corollary 3.8

For $i,j,y\in \{1,2\}$ let $\mu _{ijy}\in [0,1]$, such that the stochastic matrix *Q* defined asis irreducible and aperiodic, and denote by *q* its stationary distribution. Then,

$$\begin{aligned} \varphi _Z \;\ge \; \sum _{i,j,y,z\in \{1,2\}} q_{iy} Q^{iy,jz} \Big (\log \big (A(i,j)^{y,z}\big ) + h(\mu _{ijy})\Big ), \end{aligned}$$

where $h(x)=0$ if $x\in \{0,1\}$ and $h(x)=-x\log (x)-(1-x)\log (1-x)$ otherwise.

Let us point out that this result can also be deduced directly from Arnold et al. (1994, Theorem 4.3), where the authors, based on concepts from equilibrium statistical mechanics, established a variational characterization of the maximal Lyapunov exponent for general ergodic sequences of positive matrices satisfying certain integrability conditions. Nevertheless, for the sake of being self-contained we provide a proof of Corollary 3.8 at the end of this section. A similar upper bound has been derived by Gharavi and Anantharam (2005).

Note that Corollary 3.8 in this special case offers an analytical approach for finding an over-all reliable lower bound by adjusting the eight $\mu$-parameters. Additionally, it provides a way to give an approximate uniform lower bound, which, in some regimes, outperforms the other lower bounds discussed here—see Fig. 9 (mid).

#### Remark 3.9

*(Connection to* Hautphenne and Latouche 2016*and* Kussell and Leibler 2005*).*

1. Using Eq. (3.5) for both mean matrices of the stochastic switcher yields $A(i,j)=M(i)$. Hence, letting $\lambda (e,\cdot ) = \varrho (e)$, Proposition 3.6-(a) gives the same upper bound as in Hautphenne and Latouche (2016, Theorem 2). Changing the values of $\lambda$ allows to influence the loss from the estimation by Jensen’s inequality, offering potential improvement for this upper bound.
2. Analogous to Corollary 3.8 the method in Hautphenne and Latouche (2016) is based on constructing $\nu _n$ via transition matrices of the form
  $$\begin{aligned} \Theta (e) \;\mathrel {\mathop :}=\; {{\,\mathrm{\mathrm {diag}}\,}}(v(e))^{-1} \cdot \frac{M(e)}{\varrho (e)} \cdot {{\,\mathrm{\mathrm {diag}}\,}}(v(e)), \end{aligned}$$
  where *v*(*e*) denotes the respective and suitably normalized right-eigenvectors of *M*(*e*). In fact, the lower bound in Hautphenne and Latouche (2016, Theorem 3) can be achieved from Corollary 3.8 by choosing $\mu _{ijy} = \Theta (i)_{y1}$ and, as above, $A(i,j)=M(i)$ by decomposition (3.5). Then, the lower bound in Corollary 3.8 becomes
  3.10 $$\begin{aligned} {{\,\mathrm{{\mathbb {E}}}\,}}[\log \varrho (0)] + q \bigg ( {\mathbb {I}}_4 - \begin{pmatrix} \Theta (1) &{} 0 \\ 0 &{} \Theta (2) \end{pmatrix} \bigg ) \begin{pmatrix} \log v(1)_1 \\ \log v(1)_2 \\ \log v(2)_1 \\ \log v(2)_2 \end{pmatrix}, \end{aligned}$$
  which illustrates the connection. Figure 9 demonstrates that there are choices for the parameters $\mu$ that can be made to improve the lower bound from Hautphenne and Latouche (2016), especially for small $\alpha$. This particular choice of transition matrices $\Theta (e)$ defines a Markov chain $(Y_k)$ closely related to the so-called *retrospective process* (cf. Wang 2014, Chapter 3).
3. Kussell and Leibler (2005) approximate the maximal Lyapunov exponent under a *slow environment condition*. The soundness of this approximation can be verified by the previously discussed bounds of Hautphenne and Latouche (2016): As $s_1, s_2 \rightarrow 0$ and $s_1/s_2 \rightarrow \tau >0$, $P_I$ gets close to ${\mathbb {I}}_2$ and hence, the second addend in (3.10) approaches $q({\mathbb {I}}_4-Q)\log v=0$. On the other hand, the ${\widehat{A}}^*$-matrix obtained in Remark 3.9-(1) tends to
  $$\begin{aligned} \begin{pmatrix} M(1)/\varrho (1) &{}\quad 0 \\ 0 &{}\quad M(2)/\varrho (2) \end{pmatrix} \end{aligned}$$
  and $\varrho ({{\widehat{A}}}^*) \rightarrow 1$. Thus, both bounds approach ${{\,\mathrm{{\mathbb {E}}}\,}}[\log \varrho (0)]$ and so does $\varphi _Z$.

#### Proof of Corollary 3.8

Denote by $\gamma _i = \frac{q_{i1}}{q_{11}+q_{12}}$ and by $(Y_k)_{k \ge 1}$ random variables on $\{1,2\}$ holding ${{\,\mathrm{{\mathbb {P}}}\,}}[Y_1=1 \mid I_1] = \gamma _{I_1}$ and

$$\begin{aligned} {{\,\mathrm{{\mathbb {P}}}\,}}[Y_{n+1} = 1 \mid Y_n, I_n, I_{n+1}] \;=\; \mu _{I_n,I_{n+1},Y_n} \end{aligned}$$

for all $n \ge 1$. Then, $((I_n,Y_n))_n$ is a time-homogeneous stationary Markov chain with transition matrix *Q*. Furthermore, $({\mathbb {Y}}_n)_n$ with ${\mathbb {Y}}_n \mathrel {\mathop :}=((I_n,Y_n),(I_{n+1},Y_{n+1}))$ is a homogeneous Markov chain with stationary distribution $q^{(2)}$ given by

$$\begin{aligned} q^{(2)}_{ab} \;=\; q_a\cdot Q^{ab} \;=\; ({{\,\mathrm{\mathrm {diag}}\,}}(q)\cdot Q)^{ab} \end{aligned}$$

for $a, b \in \{11, 12, 21, 22\}$. Now, $X_n = f({\mathbb {Y}}_n)$ with $f(i,y,j,z) = \log \big (A(i,j)_{y,z}\big )$. Letting $\nu _n^I$ the distribution of $(Y_k)_{1 \le k \le n}$ conditional on $(I_k)$, it follows by stationarity of the ${\mathbb {Y}}_k$ and ergodicity that

$$\begin{aligned} \frac{1}{n} \sum _{k=1}^{n-1}{\mathbf {E}}_{\nu _n^I}[X_k] \;=\; \frac{1}{n}\sum _{k=1}^{n-1}{{\,\mathrm{{\mathbb {E}}}\,}}[f({\mathbb {Y}}_k)\mid I_k,I_{k+1}] \;\xrightarrow {n \rightarrow \infty }\; {{\,\mathrm{{\mathbb {E}}}\,}}_{q^{(2)}}\!\big [ f(I_1, Y_1, I_2, Y_2)\big ] \end{aligned}$$

amounting to the first addend of the lower bound. On the other hand, using the Markov property,

$$\begin{aligned} \frac{1}{n} H(\nu _n^I)&\;\mathrel {\mathop :}=\; -\frac{1}{n} \sum _{\alpha \in \{0,1\}^n} \nu _n^I(\alpha ) \log \nu _n^I(\alpha ) \;=\; -\frac{1}{n} {{\,\mathrm{{\mathbb {E}}}\,}}\!\big [\log \nu _n^I(Y_1, \ldots , Y_n) \mid (I_k)_{k \le n}\big ]\\&\;=\; \frac{1}{n} \sum _{k=1}^{n-1} {{\,\mathrm{{\mathbb {E}}}\,}}\!\big [ h(\mu _{I_kI_{k+1}Y_k}) \mid I_k,I_{k+1} \big ] + O(1/n). \end{aligned}$$

Hence, by stationarity of $({\mathbb {Y}}_k)$ and the Ergodic Theorem it follows

$$\begin{aligned} \frac{1}{n} H(\nu _n^I) \;\xrightarrow {n\rightarrow \infty }\; {{\,\mathrm{{\mathbb {E}}}\,}}_{q^{(2)}}\!\big [h(\mu _{I_1I_2Y_1})\big ] \end{aligned}$$

and the corollary holds by Proposition 3.6-(b). $\square$

## Discussion and outlook

### Discussion

In the previous sections we evaluated and compared the fitness of 2-type branching processes with dormancy. We incorporated different switching strategies between active and dormant states, type-specific relative reproductive costs and fluctuating environments changing between healthy and harsh states. We will now discuss our results from a somewhat elevated perspective, formulating simplified ‘take-home messages’, and put them in the context of earlier work. Further, we will comment on the role of stochasticity in our model and sketch areas for future research.

### ‘Take-home messages’ and the ‘rules of thumb’ of Malik and Smith

In Malik and Smith (2008), the authors work in a dynamical-systems based set-up and provide several ‘rules of thumb’ summarizing their findings. We try to formulate a corresponding set of such rules; however, one should be careful with these necessarily vague statements—in doubt one should always refer to the exact mathematical results. The following statements hold under our ‘fair comparison’ assumption ($\gamma =1$).

(1) Each switching strategy can be more fit than the ‘sleepless’ strategy when i) good times are rare and ii) bad times are sufficiently harsh. Otherwise, the sleepless population has a (potentially strong) fitness advantage.

This was essentially also observed in Malik and Smith (2008). Here, i) corresponds in our setting to the condition $s_2\ll s_1$. In this case indeed we see that $\varphi _X$ is small—cf. Fig. 3. However, one should note that this observation also depends on the severity $\alpha$ of the ‘harsh’ environmental state, as $\varphi _X$ will eventually always dominate as $\alpha \rightarrow 1$, thus explaining the additional condition ii). In fact, we can strengthen this rule by adding that each switching strategy can even be exclusively super-critical (“strong fitness advantage”), if the ratio between good times and bad times is sufficiently adjusted, cf. the colored areas of Fig. 3. From now on, we will always assume a sufficiently severe harsh environment (that is, $\alpha$ is sufficiently small).

Note that in the opposite limit $\alpha \downarrow 0$ the horizontal line in Fig. 3 tends to a horizontal line through the point 1, the slope of the linear function through the origin tends to $\infty$, and the separatrix between the responsive and spontaneous switcher tends to the function $[0,1] \ni s_1 \mapsto 1-\mathbbm{1}_{(0,1]}(s_1)$. In this case, the stochastic switcher completely dominates the diagram, but has no strong fitness advantage.

(2) The responsive switcher is more fit than the stochastic switcher when environmental states change rarely, i.e. when $s_1\cdot s_2$ is small.

Note that a similar rule has been stated in Kussell and Leibler (2005). The corresponding rule in Malik and Smith (2008) is that the “responsive switcher is more fit than the stochastic switcher when either good times are very rare or are very common”. In our case, this would correspond to the condition that either $s_1 \ll s_2$ or $s_2 \ll s_1$, suggesting that the point $(s_1,s_2)$ lies below the graph of a suitable ‘hyperbola’. Indeed, we are able to compute the exact boundary of the region where $\varphi _{\mathrm {res}}>\varphi _{\mathrm {sto}}$, which is given by (2.10), see Fig. 3 and Remark 2.22. Hence we are able to provide a very explicit classification of fitness advantage areas. However, we also see that $\varphi _{\mathrm {res}}>\varphi _{\mathrm {sto}}$ when environmental states *both* change rarely, i.e. also if $s_1$ and $s_2$ are small. Since Malik and Smith (2008) only consider environmental cycles of fixed length $T\equiv s_1^{-1}+s_2^{-1}$, they cannot observe this effect. In contrast, Malik and Smith (2008) also provide results for the limit of extremely quickly fluctuating environments, which are meaningless in our model, since we assume discrete time/generations.

(3) When the environment changes almost every generation, i.e. when $s_1\cdot s_2$ is large, the preliminary switcher is the fittest.

The preliminary switcher does not appear explicitly in Malik and Smith (2008) and might seem counter-intuitive at first glance. However, in the discrete generation context, when the environment becomes predictable (e.g. seasonal/periodic changes), this strategy can be optimal, as Fig. 3 shows.

(4) For intermediate values of $s_1\cdot s_2$, stochastic switching emerges as optimal strategy.

As discussed in Remark 2.21, stochastic switching can be constructed via convex combinations of responsive and preliminary switching, retaining rank 1. Their Lyapunov exponents, $\varphi _{\text {cc}}(q(1),q(2))$, can then be computed by Theorem 2.14 and maximized in *q*(1) and *q*(2) to obtain the optimal rank-1-strategy under fair comparison, which is a non-trivial convex combination for intermediate $s_1\cdot s_2$ as Fig. 4 suggests. Indeed, mixed strategies can be uniquely super-critical (‘strong fitness advantage’). This observation has no analogue in Malik and Smith (2008), where although ‘hybrid’ strategies are mentioned (p. 1144), no results are being provided. However, in general the optimal strategy might not be of rank 1, as Fig. 7 suggests that non-zero determinants might further increase fitness in certain scenarios.

### The model of Dombry, Mazza and Bansaye

In Dombry et al. (2011), the authors deal with a branching process model for *phenotypic switches* between potentially many types, and with a general class of random environments (though still assumed to be stationary and ergodic). However, in some regards their model and results are also more restrictive than ours, and some scenarios of dormancy-related reproduction are not covered. To understand the differences, let us recall their distinction between *hereditary* and *non-hereditary* reproduction strategies. In non-hereditary strategies, in a first step, the offspring numbers of individuals are sampled, and then, in a second step, *independently*, the new phenotypes are attached to the offspring individuals. This case disentangles reproduction and phenotype-allocation and allows to obtain a wealth of elegant results on the fitness and optimality of switching strategies. In contrast, the hereditary case does not feature this disentanglement, and type allocation may depend on the type of the parents. Because of this, this case is mathematically much harder to investigate. In fact, here, Dombry et al. (2011) provide no systematic results for the Lyapunov exponents of the system.

Unfortunately, the non-hereditary case already excludes our simple example for dormancy-related reproduction being the result of either binary fission or sporulation from Sect. 2.1, since here, the offspring number determines the offspring type. It is still possible to transfer some of their machinery to the cases which in our models correspond to rank-1 matrices, but not to the rank-2 case.

Nevertheless it is interesting to compare some of the results for the non-hereditary case with our results. In the case with spontaneous switching (related to the ‘no-sensing’ case in the language of Dombry et al. (2011)), they show that there are situations where a diversified strategy with several phenotypes can have a strong fitness advantage over a ‘single-type’ strategy, cf. their Section 1.1.1. For the case ‘with sensing’ (responsive switching) and for $s_1=s_2$, they show that for rarely changing environments, responsive switching is optimal, whereas in highly fluctuating environments, preliminary switching dominates. Further, a mixed strategy—e.g. stochastic switching—is optimal in intermediate regimes. This corresponds to our observation in Fig. 3 (left). However, similarly to the iid case given on the line where $s_1=1-s_2$, this covers only a small part of the fitness landscape.

### The role of relative reproductive costs and ‘weighted fair comparison’

Note that while (Dombry et al. 2011) do consider mixed switching strategies, they implicitly always assume a ‘fair-comparison’ of reproductive strategies (cf. the fixed type distributions $\Upsilon _{t,e}$ on p. 377), corresponding to our comparison with $\gamma = 1$. If this assumption is violated, that is, dormant offspring are either ‘more cost efficient’ than active offspring ($\gamma < 1$), or ‘more expensive’ ($\gamma > 1$), the picture regarding optimal strategies becomes very rich and exhibits novel effects. Note that situations in which dormant offspring are more expensive than active offspring could relate for example to the sporulation process of Bacillus subtilis, which takes much longer than producing an active offspring by binary fission (Piggot and Hilbert 2004), and thus leads to fewer dormant offspring per time unit, resulting in higher ‘effective’ costs. On the other hand, plants often produce many seeds at a low cost, and this is clearly the optimal strategy in the presence of extremely harsh environments (‘winter’) that effectively kills all ‘active’ individuals from the current generation of a species.

In our model, the following picture emerges (cf. Fig. 5): Under reduced costs for dormant individuals (here, $\gamma = 1/2$), rule (1) still holds in a qualitative sense; however, the region where the sleepless case is optimal is further reduced, while the relation between the regions of the switching strategies does not seem to change qualitatively. Under increased costs for dormant offspring (here, $\gamma = 2$), a new effect appears. Here, the ‘sleepless’ population can suddenly gain a strong fitness advantage in moderately fluctuating environments, at the cost of the stochastic switcher.

Interestingly, both the responsive and the preliminary strategies seem less severely affected by variable relative reproductive costs as both retain their ‘strong advantages’ in Fig. 5 (right). However, for severe relative fitness differences, the qualitative picture may again change drastically. For example, in Fig. 6 (left) the separatrix between the regions of dominance of the responsive and the stochastic switcher performs a phase transition, where for $\gamma =1/9$ it becomes a straight line. This invites a more comprehensive study of the sensitivity of the optimal strategies on the relative reproductive costs, which however seems beyond the scope of the present paper.

### Stochastic versus deterministic modeling

Stochasticity enters in our model in two distinct ways. The first is environmental stochasticity, where random changes between harsh and healthy states affect all individuals in the population simultaneously. Second, there is demographic stochasticity, due to random reproduction/switching (described by a branching process), which is independent between individuals. Despite these two sources of randomness, a first observation is that several qualitative results remain valid under both approaches (e.g. rule 1).

One reason is that the reproductive mechanism enters our results on the maximal Lyapunov exponents only through the mean matrices of the offspring distributions, which yields the same information as is contained in the transition rates of dynamical systems. Further, for large populations, the law of large numbers implies that branching processes in discrete time, resp. birth-death processes in continuous time, can be well approximated by dynamical systems, see e.g, Kurtz (1974), Ethier and Kurtz (1986) and Fournier and Méléard (2004) (the latter for spatial and measure-valued set-ups) on the ecological time-scale.

However, demographic stochasticity does play a major role in situations when population sizes may fluctuate strongly, and in particular may be very small. This could for example be the case in scenarios when new dormancy traits invade a resident population without this trait (as in Blath and Tobiás 2020), or infections in an early stage. It is certainly also relevant when considering extinction probabilities, which by definition involve small population sizes. See e.g. Jost and Wang (2014), who investigate the extinction probability of branching processes under optimal phenotype allocation.

Environmental stochasticity determines the ‘random order’ in which the mean matrices corresponding to the environmental states enter the formula for the Lyapunov exponent. The formula of course also holds for deterministic periodic environments. In the rank-1 case, exponents can e.g. be computed via Lemma 3.1, and this can lead in (suitably chosen) environments to similar effects as in corresponding random environments (e.g.  a periodic environment, where two harsh periods are always followed by two healthy periods and vice versa, and a random environment with $s_1=s_2=1/2$). However, in the (non-commutative) rank two case, the relation between stochastic and deterministic environments, and the impact on optimal switching strategies, is not clear and requests future work.

### Outlook

As indicated above, our results are still incomplete and our study invites further research in several directions.

For example, progress regarding the exact computation of Lyapunov exponents is certainly desirable, but this is known to be difficult and probably needs particular and sophisticated methods for particular switching strategies, depending on the algebraic properties of the underlying mean matrices. This also holds true for more general environments. An alternative to analytic solutions could be the exploration of the ‘strategy space’ via simulation, e.g. involving genetic algorithms. Simulation methods in more complex models could also allow the assessment of antibiotic treatment protocols.

A readily accessible task is the extension of our model to continuous-time birth-death processes. This then suggests to explore the relation to ‘adaptive dynamics’ related set-ups, in which one could try to merge random environments and dormancy with competition and mutation. A starting point could be the recent model on dormancy under competition in Blath and Tobiás (2020), extended by rates depending on the state of a fluctuating environment. Having more than one species with potentially different dormancy strategies, perhaps even in a spatial set-up à la (Fournier and Méléard 2004), could lead to truly ecological models. However, this would also require to disentangle different notions of fitness (e.g. invasion fitness vs. long-term fitness).

Finally, such general models could form the basis to understand effects of long-term changes in the distribution of the random environment (for example due to climate change). It is well-known that climate change can have a serious impact on seed banks, see e.g. Ooi (2012), and it would be interesting to understand the robustness of switching methods under such scenarios.

## Appendix

### Proof of Proposition 2.2

For the classical BGW process $(X_n)$ with offspring distribution $Q_X$ we write $h\!: [0,1] \rightarrow [0,1]$, $s \mapsto h(s)={{\,\mathrm{{\mathbb {E}}}\,}}[s^{X_1} \,|\, X_0 = 1]$ to denote the corresponding offspring probability generating function. Set $h'(1)=\mu _X$ and $h(0)=Q_X(0)$. Recall that, by assumption, the variance of $Q_X$ is finite. It is well-known (cf. Athreya and Ney 1972, Theorem I.5.1), that the survival probability $\sigma _X$ is given by $1-x^*$, where $x^*$ is the smallest fixed point of $h_X$. Furthermore it is known that $(\mu _X)^{-n}{{\,\mathrm{{\mathbb {P}}}\,}}\!\big [X_n>0\big ]$ for $n\rightarrow \infty$ converges to a positive limit if $\mu _X<1$, as does $n{{\,\mathrm{{\mathbb {P}}}\,}}\!\big [X_n>0\big ]$ if $\mu _X=1$ (cf. Athreya and Ney 1972, Corollary I.11.1 and Athreya and Ney 1972, Theorem I.9.1, respectively). Since $X_n>0$ iff $T_{X}>n$, it follows that

$$\begin{aligned} {{\,\mathrm{{\mathbb {E}}}\,}}\!\big [T_{X}\big ] \;=\; \sum _{n \ge 0}{{\,\mathrm{{\mathbb {P}}}\,}}\!\big [T_{X}>n\big ] \;=\; \sum _{n\ge 0}{{\,\mathrm{{\mathbb {P}}}\,}}\!\big [X_n>0\big ] \end{aligned}$$

is infinite if $\mu _X=1$ and finite if $\mu _X<1$.

Coming to $(Z_n)$, its offspring probability generating function is given by

$$\begin{aligned} g\!: [0,1]^2 \rightarrow [0,1]^2, \qquad (s_1,s_2) \;\longmapsto \; \begin{pmatrix} {{\,\mathrm{{\mathbb {E}}}\,}}\Big [s_1^{Z_1^1}s_2^{Z_1^2} \,\Big |\, Z_0=(1,0)\Big ] \\ {{\,\mathrm{{\mathbb {E}}}\,}}\Big [s_1^{Z_1^1}s_2^{Z_1^2} \,\Big |\, Z_0=(0,1)\Big ] \end{pmatrix}. \end{aligned}$$

Now, by Athreya and Ney (1972, Theorem V.3.2), $1 - \sigma _Z$ can be given as the first component of the smallest fixed point of *g*. Denoting $w = Q_Z^2(1,0)$ and $d = Q_Z^2(0,0)$ as in the example in Sect. 2.1, basic transformations yield that $g(s_1,s_2) = (s_1,s_2)$ iff

$$\begin{aligned} \begin{pmatrix} g_1(s_1,s_2)\\ s_2 \end{pmatrix}&\;=\; \begin{pmatrix} s_1 \\ \frac{d+ws_1}{d+w} \end{pmatrix}, \end{aligned}$$

such that by monotonicity $1-\sigma _Z = g_1(1-\sigma _Z,\frac{d+w(1-\sigma _Z)}{d+w})$.

From (2.3) we obtain for $s\in [0,1]$ that

$$\begin{aligned} h(s) \;=\; 1-\sum _{k\ge 1}Q_X(k)(1-s^k) \;\le \; g_1(s,s) \;<\; g_1(s,\tfrac{d+ws}{d+w}), \end{aligned}$$

which implies that $1-\sigma _Z \ge 1-\sigma _X$, where the inequality is strict, if $\sigma _X > 0$.

It remains to prove the results regarding $T_Z$. For this, denoting by $m_1 = {{\,\mathrm{{\mathbb {E}}}\,}}[Z_1^1 \mid Z_0=(1,0)]$ and by $m_2 = {{\,\mathrm{{\mathbb {E}}}\,}}[Z_1^2 \mid Z_0=(1,0)]$, the mean matrix of the offspring distribution of *Z* is given by

$$\begin{aligned} M \;=\; \begin{pmatrix} m_1 &{}\quad m_2 \\ w &{}\quad 1-w-d \end{pmatrix} \end{aligned}$$

along with its largest eigenvalue

5.1 $$\begin{aligned} \varrho \;\equiv \; \varrho (w,d)&\;=\; \frac{1}{2}\, \Big (m_1 + 1-w-d + \sqrt{(m_1-(1-w-d))^2 + 4wm_2}\Big ) \nonumber \\&\;\ge \; \max \{m_1,1-w-d\}. \end{aligned}$$

Then, $(Z_n)$ survives with positive probability iff $\varrho > 1$, while ${{\,\mathrm{{\mathbb {P}}}\,}}\!\big [|Z_n|>0\big ]\xrightarrow {n\rightarrow \infty }0$, if $\varrho \le 1$ (cf. Athreya and Ney 1972, Theorem V.3.2). More importantly, for case *(3)*, when *d* is so small that $1-d > \mu _X$, (5.1) implies $\varrho (0,d) > \mu _X$. Thus, by continuity, there also is $w > 0$ such that $\varrho (w,d) > \mu _X$.

We now apply Athreya and Ney (1972, Theorem V.4.4): Since the offspring distributions are of finite variance, the second moment condition holds and the theorem implies

$$\begin{aligned} \lim _{n\rightarrow \infty } \varrho ^{-n} {{\,\mathrm{{\mathbb {P}}}\,}}\!\big [Z^1_n+Z^2_n>0\big ] \;\in \; (0,\infty ), \end{aligned}$$

which concludes the proof of (*3*).

For the critical case (*2*) note that $\varrho \le \max \{m_1+m_2,1-d\}$. (The maximum row sum can be seen as an operator norm and hence is an upper bound for all eigenvalues.) Hence, in the case $m_1 + m_2 < \mu _X = 1$ the proof is complete, since ${{\,\mathrm{{\mathbb {P}}}\,}}[T_Z > n] \approx \varrho ^n$. At last, it remains to show that even if $m_1 + m_2 = 1$, $\varrho < 1$. Thus, letting $m_1 = 1-m_2$ note that $\varrho = 1$ iff

$$\begin{aligned} \sqrt{(w+d-m_2)^2+4wm_2} \;=\; m_2 + w+d, \end{aligned}$$

which can only hold if either $d=0$ or $m_2=0$, both contradicting our assumptions. $\square$
